# Assessment of the National Park network of mainland Spain by the Insecurity Index of vertebrate species

**DOI:** 10.1371/journal.pone.0197496

**Published:** 2018-05-21

**Authors:** Alba Estrada, Raimundo Real

**Affiliations:** 1 Biogeography, Diversity and Conservation Research Team, Department of Animal Biology, Universidad de Málaga, Málaga, Spain; 2 Research Unit of Biodiversity (UMIB, UO-CSIC-PA), Oviedo University–Campus Mieres, Mieres, Spain; 3 Instituto de Investigación en Recursos Cinegéticos—IREC (CSIC-UCLM-JCCM), Ciudad Real, Spain; Universidad de Sevilla, SPAIN

## Abstract

The evaluation of protected area networks on their capacity to preserve species distributions is a key topic in conservation biology. There are different types of protected areas, with National Parks those with highest level of protection. National Parks can be declared attending to many ecological features that include the presence of certain animal species. Here, we selected 37 vertebrate species that were highlighted as having relevant natural value for at least one of the 10 National Parks of mainland Spain. We modelled species distributions with the favourability function, and applied the Insecurity Index to detect the degree of protection of favourable areas for each species. Two metrics of Insecurity Index were defined for each species: the Insecurity Index in each of the cells, and the Overall Insecurity Index of a species. The former allows the identification of insecure areas for each species that can be used to establish spatial conservation priorities. The latter gives a value of Insecurity for each species, which we used to calculate the Representativeness of favourable areas for the species in the network. As expected, due to the limited extension of the National Park network, all species have high values of Insecurity; i.e., just a narrow proportion of their favourable areas are covered by a National Park. However, the majority of species favourable areas are well represented in the network, i.e., the percentage of favourable areas covered by the National Park network is higher than the percentage of mainland Spain covered by the network (result also supported by a randomization approach). Even if a reserve network only covers a low percentage of a country, the Overall Insecurity Index allows an objective assessment of its capacity to represent species. Beyond the results presented here, the Insecurity Index has the potential to be extrapolated to other areas and to cover a wide range of species.

## Introduction

**Fig 1:**
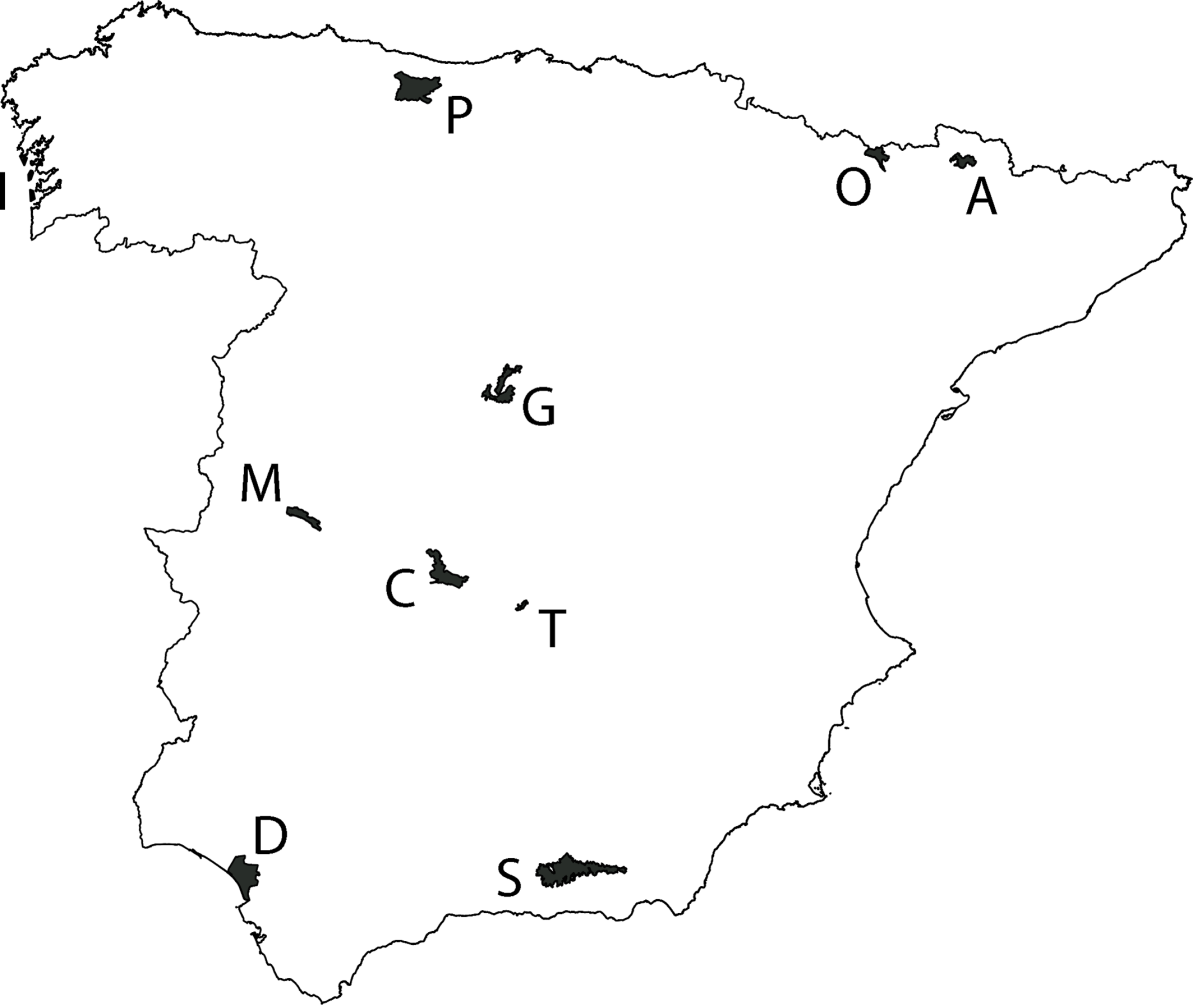
National Park network of mainland Spain. I: Islas Atlánticas de Galicia, P: Picos de Europa, O: Ordesa y Monte Perdido, A: Aigüestortes i Estany de Sant Maurici, G: Sierra de Guadarrama, M: Monfragüe, C: Cabañeros, T: Tablas de Daimiel, D: Doñana, S: Sierra Nevada.

Protected areas are essential in biodiversity conservation [1]. Although the pristine aims for the declaration of a protected area may differ, e.g. the protection of the scenery or landscape [2] or the protection of particular species (EU Birds Directive 2009/147/EC), the benefits of the declaration goes beyond this initial intention and, in general, all the wildlife inhabiting a protected area enjoy higher protection than populations living outside the area. A country has normally different protected area networks that vary depending on the administration that declares them, having also different status of protection and restrictions. National Parks are the ones with highest level of protection. In Spain, National Parks are defined as ‘natural areas with high ecological and cultural value, little transformed by exploitation or human activity which, due to the beauty of their landscapes, the representativeness of their ecosystems or the singularity of their flora, fauna, geology or geomorphological formations, possess outstanding ecological, aesthetic, cultural, educational and scientific values whose conservation deserves a preferential attention and are declared of general interest of the State’ [3]. The National Park network of Spain was established a century ago (in 1916), and nowadays it is formed by 15 protected areas, being 10 of them in mainland Spain (Fig 1).

The assessment of protected area networks on how well they cover the distribution of biodiversity is called gap analysis and was first defined in the early nineties by Scott et al. [4]. A key point in the process is to define what we meant with biodiversity, i.e., it is required to select surrogates of biodiversity. Setting targets for protected-area planning is not a trivial task, and because gap analysis is a spatial exercise, only biodiversity features that can be mapped are of practical value for this purpose [1]. Gap analyses have been performed taking into account different surrogates or conservation-value criteria, such as, the distribution of particular species [5], species richness [6], rarity [7], vulnerability [8], endemicity [9], species abundance [10], minimum area to support viable populations [11], maintenance of patterns and processes [12], ecosystem representativeness [13], supply of ecosystem services [14], or a combination of indexes [8, 9, 15]. Additionally, studies have taken into account actual distributions of species or their potential distributions [16, 17], even under land use [18, 19] and/or climate change scenarios [20–22]. Species distributions are normally recorded in atlases that use grids of cells; thus, another important decision is to select a threshold to define a cell as protected. For example, in order for a 10 km × 10 km cell to be considered protected, it may be required to be covered at least in a 25% by a protected area [16]. As expected, the threshold chosen has implications on the obtained results [23] (but see [5]).

Díaz-Gómez et al. [24] proposed the Insecurity Index as a measure of species protection gap to be used in gap analysis. The Insecurity Index is based on species distribution modelling and fuzzy logic. One of its advantages is that it avoids the use of a threshold to decide the protection status of a cell. In contrast, it uses the percentage of the cell that it is protected, which is an objective metric to classify the protection status of the cells [25]. Regarding biodiversity, the Insecurity Index takes into account the favourability of the species in each of the cells [26]. Therefore, it also avoids a threshold to define the distribution of the species (presence/absence or suitable/unsuitable). Building the index on favourable areas allows the identification of the relevant areas for the species, i.e., not only where the species is present nowadays but also where the conditions are appropriate for the species even in the absence of it. When applied to many species, favourability allows to take into account both observed and dark diversity. “Dark diversity” is defined as the pool of absent species that theoretically could inhabit a suitable region [27], it is a valuable indicator in nature conservation [28] and can be applied to design and/or prioritize nature reserve networks [29–31]. Thus, with favourability we focus the attention on the locations where the species has a greater or lesser potential to be observed [32]. Favourability represents the complete information about the species potential presence, provided that the model has succeeded in capturing the relevant predictors of the species distribution. In this sense, individuals representing any species would then not be assumed to be inside a protected area or otherwise; rather, they would be treated as having some relative likelihood of occurring within the area, which requires the application of fuzzy logic. Here, we apply the Insecurity Index to the National Park network of mainland Spain, in order to evaluate if favourable areas of some vertebrate species, which are considered relevant to this network, are well represented in it.

## Materials and methods

### National Park network

We downloaded the shapefile of the National Park network of Spain from the webpage of the Spanish Ministry of the Environment (http://www.magrama.gob.es/es/red-parques-nacionales/sig/). The vector data on protected areas (Fig 1) was intersected with the vector UTM projected grid of mainland Spain at a resolution of 10 km x 10 km. For each cell of the UTM grid, we calculated the proportion of the cell covered by protected areas (*P*_j_). *P*_*j*_ ranges between 0 and 1, with a cell receiving a value of 1 when is completely covered by protected areas.

### Modelling method

We chose species that were highlighted as having relevant natural value for at least one of the 10 National Parks of mainland Spain, as it was detailed in the description of the park (http://www.mapama.gob.es/es/red-parques-nacionales/). The number of species selected was 37 and included taxa of the four terrestrial vertebrate groups, i.e., amphibians, reptiles, mammals and birds (Table 1). We used presence/absence in mainland Spain in each 10 km x 10 km UTM cell according to the distribution atlases of vertebrates in Spain [33–35]. In the case of birds, distribution referred to breeding sites.

**Table 1:** Species analysed in the present study.

Group	Species	IUCN	Reference	National Park
Amphibians	*Chioglossa lusitanica*	VU	[34]	P
	*Euproctus asper*	NT	[34]	A, O
	*Pleurodeles waltl*	NT	[34]	D, M, T
	*Bufo calamita*	LC	[34]	S, D, M, T
	*Hyla meridionalis*	NT	[34]	M, S
	*Rana iberica*	VU	[34]	G
	*Rana pyrenaica*	VU	[34]	O
Reptiles	*Emys orbicularis*	VU	[34]	T, D, C
	*Mauremys leprosa*	VU	[34]	M, S
	*Testudo graeca*	EN	[34]	D
	*Anguis fragilis*	LC	[34]	P, A, I
	*Podarcis muralis*	LC	[34]	G, A
	*Elaphe scalaris*	LC	[34]	S, I
Birds	*Phalacrocorax aristotelis*	VU	[36]	I
	*Ciconia nigra*	VU	[36]	G, C, M, D, T
	*Netta rufina*	VU	[36]	T, D
	*Gypaetus barbatus*	EN	[36]	A, O
	*Neophron percnopterus*	EN	[36]	M, D, O, P, C
	*Aegypus monachus*	VU	[36]	G, C, M, D
	*Aquila adalberti*	EN	[36]	D, G, C, M
	*Aquila chrysaetos*	NT	[36]	A, O, C, M, S, P
	*Lagopus mutus*	VU	[36]	A, O
	*Tetrao urogallus*	EN	[36]	P, A, O
	*Perdix perdix*	VU	[36]	A
	*Otis tarda*	VU	[36]	C, D
	*Picus viridis*	LC	[36]	S, D, O, P, I, A
	*Pyrrhocorax graculus*	NE	[36]	O, P, A
	*Pyrrhocorax pyrrhocorax*	NT	[36]	A, S, O, P, I, M
Mammals	*Galemys pyrenaicus*	VU	[35]	A, P
	*Canis lupus*	NT	[35]	P
	*Lutra lutra*	NT	[35]	M, D, G, P, I, C, T, A
	*Ursus arctos*	CR	[35]	P, A
	*Felis silvestris*	VU	[35]	G, M, D, P, C, S, A
	*Lynx pardinus*	CR	[35]	D
	*Cervus elaphus*	VU	[35]	C, D, P, M, A
	*Rupicapra pyrenaica*	LC	[35]	A, O, P
	*Capra pyrenaica*	VU	[35]	S

IUCN is the threat status of the species in Spain according to distribution atlases and red books of vertebrates [34–36]. CR: critically endangered, EN: endangered, VU: vulnerable, NT: near threatened, LC: least concern. The column National Park indicates those parks that highlight the species as having relevant natural value or that mention the species in the description of the park. Note that this column does not collect all the parks with presence of the species. P: Picos de Europa, A: Aigüestortes i Estany de Sant Maurici, O: Ordesa y Monte Perdido, D: Doñana, M: Monfragüe, T: Tablas de Daimiel, S: Sierra Nevada, G: Sierra de Guadarrama, C: Cabañeros, I: Islas Atlánticas de Galicia (see Fig 1).

To model the distribution of each species, we considered a pool of environmental variables related to five predictor sets, i.e., climate, topography, lithology, land use, and human activity (S1 Table). The number of variables, especially within the climate predictor set, was large and many of them showed high values of correlation. Thus, for each species and predictor set, we calculated pairwise correlations between the variables and, among those pairs of variables with a Pearson correlation value above 0.8, we excluded the one with the least significant individual relationship with the distribution of the species. Additionally, a spatial descriptor was added to the list of variables in the modelling process to account for spatial autocorrelation. This descriptor was obtained following the ‘‘trend surface approach” [37] as described in Estrada et al. [38].

Then, with the variables selected in the above mentioned procedure, we calculated the false discovery rate–FDR [39] to control for type I errors. We only accepted those variables that were significantly related to the distribution of the species under a FDR of q < 0.05. Finally, we performed a forward-backward stepwise logistic regression based on the Akaike Information Criterion (AIC) on the variables that were retained in the FDR test. From the logistic regression model, we obtained favourable areas for each species in Spain after applying the favourability function proposed by Real et al. [26]:

$$F=\frac{e^{y}}{\frac{n_{1}}{n_{0}}+e^{y}}$$

where *e* is the basis of the natural logarithm, *y* is the logit function of the logistic regression model, *n*_*1*_ is the total number of presences, and *n*_*0*_ is the total number of absences. The favourability function reflects the degree (between 0 and 1) to which the local probability values differ from that expected according to the species prevalence, where *F* = 0.5 corresponds to *P* = prevalence. Probability depends both on the response of the species to the predictors and on the overall prevalence of the species [40], while favourability values only reflect the response of the species to the predictors [41]. The use of the favourability function allows the application of fuzzy logic to the resulting spatial analysis of the species [9, 16, 42], e.g., the calculation of the Insecurity Index (see below).

We assessed the classification power of the models by calculating Cohen’s kappa, sensitivity, specificity, and their Correct Classification Rate (CCR), using the favourability value of *F* = 0.5 as classification threshold, and evaluated the discrimination capacity using the Area Under the Curve (AUC) of the Receiver Operating Characteristic, which is independent of any favourability threshold. The goodness-of-fit of the models was assessed using the Hosmer and Lemeshow test (HL) for 10 bins of probabilities (each one with a range of 0.1) [43].

All analyses were performed in R [44] with the packages *fuzzySim* [45, 46] and *modEvA* [47]. We produced maps of the predictions with the package *maptools* [48], and represented them in QGIS [49].

### Calculation of the Insecurity Index

Díaz-Gómez et al. [24] defined the Insecurity Index of species based on species distribution modelling and fuzzy logic. The Insecurity Index (0–1) of a species represents how much of the fuzzy set of favourable areas for the species is not included in protected areas. The larger the extent of favourable areas of a species that is not covered by protected areas, the higher the Insecurity Index. Two metrics of Insecurity Index are defined for each species: the Insecurity Index in each of the cells (*I*_*ij*_), and the Overall Insecurity Index of a species (*I*_*i*_).

The Insecurity Index in each of the cells is defined as:

$$I_{ij}=F_{ij}-(F_{ij}*P_{j})$$

where *I*_*ij*_ is the Insecurity Index of the species *i* in the cell *j*, *Fij* is the favourability for the species *i* in the cell *j*, and *Pj* is the extent of a cell that is covered by a protected area. As the index considers the percentage of the cell that it is protected, it avoids the subjective selection of a threshold to decide if a cell is protected or not [16, 23].

Mapping *I*_*ij*_ we identified insecure areas for each species that can be used to establish spatial conservation priorities. We also summarized the results for each taxonomic group by adding the *I*_*ij*_ values of all the species in a group and normalizing them, by dividing by the maximum value in the study area.

The Insecurity Index of the species *i* over the study area (Overall Insecurity Index) is defined as:

$$I_{i}=\frac{\sum_{j=1}^{n}I_{ij}}{\sum_{j=1}^{n}F_{ij}}$$

where *I*_*ij*_ is the Insecurity Index of the species *i* in the cell *j*, *Fij* is the favourability for the species *i* in the cell *j*, and *n* is the total number of cells in the study area.

This gives a value of Overall Insecurity for each species. From this value, we obtained the Overall Security Index for each species *i* (*S*_*i*_) by calculating the complement of *I*_*i*_ (*S*_*i*_ = 1 –*I*_*i*_). The Overall Security Index represents how much of the fuzzy set of favourable areas for the species is included in protected areas, which is equivalent to calculate the proportion of favourable areas that it is covered by the National Park network.

Although the assessment of a protected area network could be done with both, the Insecurity Index in each of the cells (*I*_*ij*_) and the Overall Insecurity Index of a species (*I*_*i*_), in the case of the National Park network of Spain, the Overall Insecurity (or Security) Index is more appropriate to assess the configuration of the network. The total area of the National Park network in mainland Spain represents only a 0.667% of the area of the territory. Thus, it is not feasible to assume that the network will cover a high proportion of favourable areas of any species. We considered that a species was correctly represented by the National Park network when the proportion of favourable areas covered by the network was higher than the proportion of mainland Spain covered by the network. To obtain the Representativeness of the species in the network we divided the Overall Security Index for each species by the proportion of mainland Spain covered by the network (i.e. 0.00667). Values higher than 1 imply that the percentage of favourable areas covered by the National Park network for a given species is higher than the percentage of mainland Spain covered by the network and, thus, higher than that expected by chance. We also calculated the Representativeness of observed occurrences of the species, following the same approach as above, but considering presence or absence instead of favourability.

To test if the Representativeness of favourable areas for a species within the National Park network was significantly different from what would be expected by a random distribution of the National Parks, we performed a null model approach [25]. We randomized the cells covered by National Parks, specifically, the values of the proportion covered by the network. We calculated species’ Insecurity Index with random protected cells and then obtained the Overall Security. We repeated the approach 20 times. This allows to identify species under and over- represented by the network: when the random Securities have higher values of Representativeness than the actual Security, the species is considered as under-represented by the National Park network, and when the random Securities show lower level of Representativeness than the observed Security, it is assumed that the species is well-represented [25].

## Results

Selected species consist on a good representation of the four taxonomic groups: seven amphibians, six reptiles, fifteen birds, and nine mammals (Table 1). They also include species belonging to an array of threat status, being the majority of them classified as Vulnerable in Spain according to the IUCN (International Union for Conservation of Nature) criteria (Table 1).

Variables included at each step of the modelling process (i.e., pairwise correlations, spatial descriptor, FDR, and forward-backward stepwise) for each species can be seen in S1 Appendix. Variables that formed part of the final favourability models and their coefficients, as well as evaluation metrics of the models, are also shown in S1 Appendix. Favourability models had good discrimination and classification capacity and goodness-of-fit [average and range values for the 37 species: AUC: 0.921 (0.689–1), kappa: 0.489 (0.154–0.905), sensitivity: 0.895 (0.661–1), specificity: 0.849 (0.579–0.999), CCR: 0.856 (0.639–0.999), HL: 20.936 with p: 0.212 (0.903–73.930 with p: 0–0.963)].

**Fig 2:**
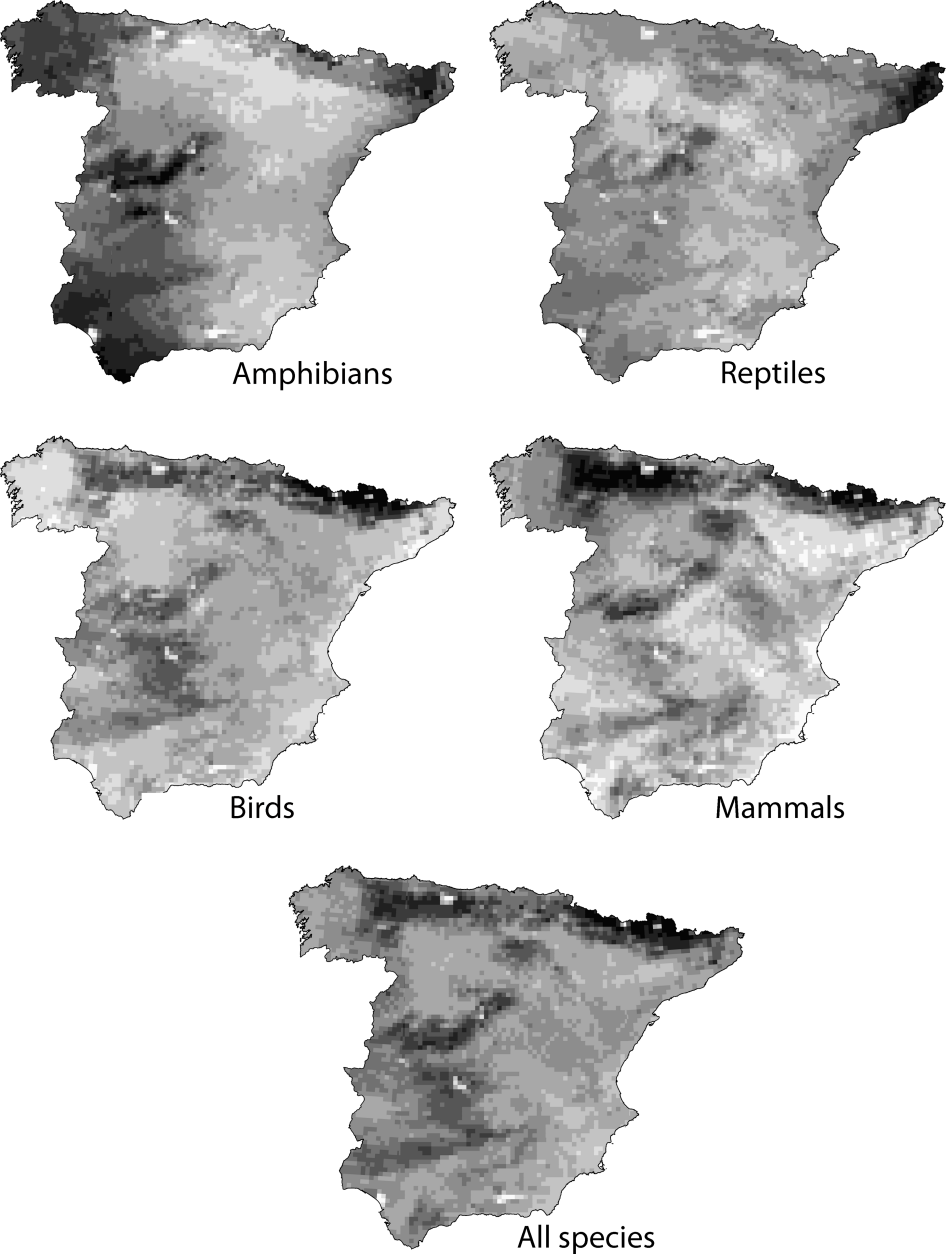
Insecurity Index in each of the cells for each taxonomic group and for all studied species. Values range from zero (white cells) to one (black cells).

Maps of the Insecurity Index for each species in each of the cells are shown in S1–S4 Figs. This maps are equal to favourability areas for each species except on the cells that are covered by National Parks. Fig 2 summarizes the Insecurity Index for each taxonomic group and for all the studied species. High values of the Insecurity Index in the maps highlight relevant areas where conservation measures could be applied. Relevant areas differ for the four taxonomic groups, being located in south-western and northern Spain in the case of amphibians, in north-eastern Spain in the case of reptiles, in the north in the case of birds, and in the north and centre of Spain in the case of mammals. Note that these relevant areas may coincide with other figures of protection that we are not evaluating in this study, such us natural parks.

Overall Insecurity and Representativeness of favourable areas for each species are detailed in Table 2. As expected, due to the limited extension of the National Park network, all species had high values of Insecurity, i.e., just a narrow proportion of their favourable areas are covered by a National Park. The amphibian *Rana pyrenaica* was the species with the highest Security, and the bird *Otis tarda* was the one with the lowest Security. Favourable areas for the majority of the species were well represented in the network, as can be deduced from the Representativeness (Table 2). Only three of the 37 species had Representativeness values lower than 1 (one reptile and two birds). Nine species had Representativeness values higher than 3, meaning that the percentage of their favourable areas covered by the National Park network is at least three times higher than the percentage of mainland Spain covered by the network. The four species with highest Representativeness were endangered species (Tables 1 and 2): an amphibian (*Rana pyrenaica*), a reptile (*Testudo graeca*) and two birds (*Lagopus mutus* and *Tetrao urogallus*). These results were consistent with those of the randomization approach: species that were better represented in the actual network than in the random networks (i.e., those that had actual Security values higher than random Securities in all cases (n = 20)), were those with the highest levels of Representativeness. On the other hand, species with lower Representativeness were the ones that had higher protection with random protected cells in more cases (Table 2). These results were consistent with the Representativeness of reported occurrences (S2 Table). We obtained high Representativeness for the majority of the analysed species: 17 species had Representativeness values of observed occurrence higher than 3, and only four species had lower Representativeness than that expected by chance (lower than 1). Three of the species with highest values of Representativeness with favourability had also the highest values with observed occurrence (Table 2 and S2 Table), and *Otis tarda* was also the species with lowest Representativeness of observed occurrence (S2 Table).

**Table 2:** Overall Insecurity Index (*I*_*i*_) and representativeness of favourable areas of species in the National Park network.

Species	Insecurity Index	Representativeness	Times actual Security > random Securities
*Chioglossa lusitanica*	0.992	1.199	11
*Euproctus asper*	0.984	2.399	20
*Pleurodeles waltl*	0.993	1.049	4
*Bufo calamita*	0.992	1.199	15
*Hyla meridionalis*	0.991	1.349	15
*Rana iberica*	0.988	1.799	20
*Rana pyrenaica*	0.875	18.741	20
*Emys orbicularis*	0.993	1.049	6
*Mauremys leprosa*	0.993	1.049	5
*Testudo graeca*	0.952	7.196	20
*Anguis fragilis*	0.991	1.349	20
*Podarcis muralis*	0.986	2.099	20
*Elaphe scalaris*	0.994	0.900	0
*Phalacrocorax aristotelis*	0.992	1.199	15
*Ciconia nigra*	0.993	1.049	10
*Netta rufina*	0.994	0.900	3
*Gypaetus barbatus*	0.981	2.849	20
*Neophron percnopterus*	0.992	1.199	14
*Aegypus monachus*	0.984	2.399	20
*Aquila adalberti*	0.982	2.699	20
*Aquila chrysaetos*	0.989	1.649	20
*Lagopus mutus*	0.951	7.346	20
*Tetrao urogallus*	0.973	4.048	20
*Perdix perdix*	0.976	3.598	20
*Otis tarda*	0.997	0.450	0
*Picus viridis*	0.992	1.199	13
*Pyrrhocorax graculus*	0.979	3.148	20
*Pyrrhocorax pyrrhocorax*	0.989	1.649	20
*Galemys pyrenaicus*	0.989	1.649	20
*Canis lupus*	0.991	1.349	18
*Lutra lutra*	0.991	1.349	20
*Ursus arctos*	0.979	3.148	20
*Felis silvestris*	0.989	1.649	20
*Lynx pardinus*	0.976	3.598	20
*Cervus elaphus*	0.991	1.349	20
*Rupicapra pyrenaica*	0.975	3.748	20
*Capra pyrenaica*	0.990	1.499	20

Representativeness is obtained after dividing the Security Index (1 –*I*_*i*_) by the ratio of mainland Spain covered by the network (i.e. 0.00667). We repeated the random Securities 20 times, so values of the third column range from 0 to 20, being 20 for well-represented species and 0 for under-represented species according to this approach.

## Discussion

The Insecurity Index has been used previously to evaluate the capacity of reserve networks to protect raptor species, both in the south of Spain [24] and in the island of Sicily (Italy) [50]. Díaz-Gómez et al. [24] obtained higher levels of insecurity, and therefore lower levels of protection, for steppe-nesting raptors than for forest- or cliff-nesting raptors. On the other hand, Sarà [50] studied the degree of protection of the steppe raptor lanner falcon (*Falco biarmicus feldeggii*) and obtained that most of its favourable areas fall outside the reserve networks. Thus, besides the declaration of new protected areas, both studies highlight the importance of agri-environmental measures for the conservation of steppe raptors. This is in agreement with studies that evaluate the protection of other steppe-bird species [15] and with the present work, as we found that the species with lowest protection of its favourable areas was the steppe bird *Otis tarda* (Table 2).

The Insecurity Index has been also proved efficient to evaluate the National Park network of mainland Spain. Although the network only covers a low percentage of the country, this method allows an objective assessment of its capacity to represent favourable areas of species. Traditional gap analyses are based on how much of the distribution of the species (or the conservation criteria used) is protected by the reserve network [4, 16]. This methodology is useful when the reserve network covers an intermediate/high proportion of the territory or when the network is widely distributed through it. None of these occurs with the Spanish National Park network, which covers a low proportion of the territory with only 10 Parks split along mainland Spain (Fig 1). Thus, a different approach was needed, being the Overall Insecurity Index (and the Representativeness derived from it), an effective tool for this purpose [24]. This also allows to rank species according to the level of Representativeness in the reserve network (Table 2), even if the network is small. It has also allowed us to evaluate the Spanish National Park network independently and not as part of other protected reserves, as was common practice in previous studies [5, 7, 8, 51]. Beyond the results presented here and in previous studies, the Insecurity Index has the potential to be extrapolated to other areas and to cover a wide range of species.

One characteristic of the Insecurity Index is that it is based on the favourability function, i.e., the aim of protection is not the presence of the species but its favourable areas [24]. Thus, if the intention is to protect observed presences of the species, another approach is needed. We have calculated the Representativeness of observed occurrence (S2 Table) but this has the problem that we cannot know if the reported presence of the species occurred inside the National Park or outside it. This drawback is diluted using the favourability values because average environmental values of the cells are considered, which take into account both the conditions outside and inside National Parks. With observed presences, all records have the same value, whereas with favourability, a range of favourability values are obtained within the distribution of the species. Favourability values inform about whether the reported presence occurred in optimal environmental conditions or not, which may be related to population density, be a sporadic or permanent presence, or occurring every year or only in some years. Using favourability for occurrence we avoided treating the species presence as a categorical data (yes or no), which entails a great loss of information and disregards the natural nuances that are actually observed. An additional intrinsic characteristic of species distributions is that they are always changing, even if known distribution data are reliable. This fact is accentuated by recent global change. Although a species were well sampled, it could have favourable areas without presences or areas with presence in suboptimal (unfavourable) conditions. The range of favourability values in areas with presence of the species are valuable, as highly favourable territories are more likely to represent source areas that may provide propagules to colonise less favourable, sink areas, in source-sink dynamics [52], thus being more valuable for conservation by the reserve network.

On the other hand, favourable areas may appear in sites where the species has not been recorded, which can be valuable as well [27]. High favourability in absent areas may reflect that the species could actually be present in that site but that it has not been recorded due, for instance, to an insufficient sampling effort [32]. But it could be also that the species is truly absent although the environment is suitable for its presence, as is the case with favourable, unoccupied patches in metapopulations. These areas may be considered potential areas to be occupied (or re-occupied in metapopulations), thus contributing to an understanding of ecological processes governing diversity patterns [27], and are valuable sites to protect [28]. This is why spatial modelling is commonly used to select or evaluate areas for species conservation [16, 20, 53].

Relevant areas for the four taxonomic groups are differently distributed through mainland Spain (Fig 2). In conservation, indicator groups are sets of species whose presence may indicate areas of high species richness [54, 55], or, in our case, areas of high favourability for many species. Our results show that there is a poor spatial overlap among relevant areas for the different vertebrate groups in Spain, which suggests that each group has its different environmental requirements, and therefore we should not select one of the taxonomic groups as surrogate to protect the others [54]. This result could be affected by the selection of the set of species, but the same pattern was found when analysing all vertebrate species by taxonomic group in the south of Spain [9]. Some of the areas highlighted as relevant for conservation by the Insecurity Index (Fig 2) coincide with other protected areas, such as Spanish natural parks, which could be elevated to National Parks if they are found to protect species of national concern that are now insufficiently protected by the National Park network.

Favourable areas for selected species are, in general, well represented in the National Park network (Table 2). Results were consistent either if the Representativeness was obtained from the Overall Security or with the randomization approach. One advantage of the first approach is that it is possible to do a ranking of the species with highest Representativeness. Some other studies have obtained a good representation or a good overlap between biodiversity distribution and reserve networks for different taxonomic groups in study areas that are located in Spain or that include this country (e.g. vertebrates [56], birds [19], raptors [57], bats [58], or lichens [17]). However, other studies have reached different conclusions and state that protected areas do not guarantee species survival of some taxa (e.g. plants [7, 20], vertebrates [8, 20, 59], herptiles [25], steppe birds [15], steppe-nesting raptors [24], or beetles [5]). Regarding the low representation of *Otis tarda* in the National Park network (Table 2), our results are in agreement with those of Traba et al. [15], who found that protected area networks were inefficient to cover the most relevant areas for steppe birds, in general, and for *Otis tarda*, in particular. This may be related to the fact that the majority of protected areas and National Parks in Spain are located in mountainous areas [17], which constitute unsuitable habitat for steppe birds [38].

Favourability models should be performed taking into account the environmental predictors that theoretically could affect the distribution of the species. Consequently, it is not recommended to use just climatic variables on the modelling process but also other predictors such us topography or land use, including a spatial descriptor [38, 60]. In this way, the output of the model will reflect the favourability for the presence of the species according to a wider set of significant causal factors. We have followed the same approach for all analysed species and have assessed a large number of causal factors with potential for affecting all of them. Alternatively, an individual, species-specific distribution model could be performed for each species using specific sets of explanatory variables. In this way, we could take into account particular predator-prey relationships [61] or other biotic interactions, such as parasites [62] or co-occurrence of parapatric species [63]. However, the inclusion of such relationships into species distribution models is not straightforward, and when analysing multiple species, some specifications may be admittedly missed in order to obtain a general pattern of favourability.

The aim of this study was to evaluate the Spanish National Park network as a whole, but some conservation implications can be derived for particular species and/or for particular National Parks. For instance, the Iberian lynx (*Lynx pardinus*) or the brown bear (*Ursus arctos*) have favourable areas in National Parks where they are absent nowadays: Cabañeros and Ordesa, respectively. Thus, if all other requirements for releases are also present (including healthy rabbit populations in the case of the lynx), these could be places to consider for future reintroductions, or these could be places where the species are expected to arrive in a natural way. Therefore, these National Parks should be aware not only of the species that are present nowadays but also of those that have the potential to be present due to the favourability of the area and that form part of the dark diversity of the Park [27].

We have obtained general high levels of Representativeness both when considering favourability (Table 2) and when considering observed occurrence (S2 Table). This does not mean that species distributions or their favourable areas are mainly concentrated in National Parks, but that the percentage of their distributions that are inside National Parks is higher than that expected by chance, i.e., the location of National Parks are well distributed through mainland Spain with regard to the distribution of the analysed species. There is a strong congruence between the Representativeness of observed occurrence or of favourable areas, but the representation is even higher for the former than for the latter (Table 2 and S2 Table). This highlights again that not all presences have the same significance within a species distribution. On the other hand, there are three species with low Representativeness of observed occurrence that has values above one if the favourability is considered (*Bufo calamita*, *Chioglossa lusitanica* and *Picus viridis*, Table 2 and S2 Table). This means that National Parks are more favourable for the presence of these species than what would be expected taking account their occurrences within them. As stated before, this constitutes valuable information for conservation of the species within National Parks. We want to note that we have calculated the Representativeness of observed occurrence for comparisons, but the Insecurity Index has only sense if applying the favourability function, for all the reasons that have been explained above and because this was the way it was originally defined [24].

The existence of protected areas is relevant for the protection of biodiversity, especially for those narrow-distributed species that occur largely within their bounds [64]. Example of these species are *Rana pyrenaica* or *Lagopus mutus* (S1 and S3 Figs), which are indeed the species with largest representation in the National Park network (Table 2). Protected areas are not only valuable for the protection of biodiversity but they are also beneficial for humans, as it has been shown that human well-being increases significantly in the presence of protected areas [65]. However, the current global trend towards human population growth and environmental degradation, implies that protected areas are becoming increasingly unconnected, like fragmented habitat islands [64]. Additionally, it is not feasible to protect a whole territory (country or region within the country) because it is materially and economically impossible [21], and because some relevant areas for biodiversity are coincident with human landscapes, such as crops [24, 38, 66] or urban areas [67]. Thus, it is essential to have biodiversity-friendly behaviours in and out protected areas. This is the only way to maintain species persistence with viable and healthy populations.

## Supporting information

S1 Table Environmental variables considered. (PDF)

S2 Table Representativeness of observed species occurrence in the National Park network. (PDF)

S1 Fig Insecurity Index for each studied amphibian in each of the cells. Values range from zero (white cells) to one (black cells).
(TIF)

S2 Fig Insecurity Index for each studied reptile in each of the cells. Values range from zero (white cells) to one (black cells).
(TIF)

S3 Fig Insecurity Index for each studied bird in each of the cells. Values range from zero (white cells) to one (black cells).
(TIF)

S4 Fig Insecurity Index for each studied mammal in each of the cells. Values range from zero (white cells) to one (black cells).
(TIF)

S1 Appendix Variables included at each step of the modelling process and final favourability models. (PDF)
