# Supplementary material for: Assessment of the National Park network of mainland Spain by the Insecurity Index of vertebrate species
Source: PLoS One. 2018 May 21;13(5):e0197496. doi: 10.1371/journal.pone.0197496 (PMC5962089; doi:10.1371/journal.pone.0197496)
Supplement: S1 Table — (PDF) [file pone.0197496.s001.pdf]

Assessment of the National Park network of mainland Spain by the Insecurity Index of vertebrate species

Alba Estrada & Raimundo Real

Plos One

S1 Table. Environmental variables considered. Variables are the average value in the 10 km x 10 km UTM cell. In the case of land use variables, the values correspond to the proportion of land covered by each land use. Sources and original resolutions are detailed under the table.

Variable	Code	Variable	Code
**Climate**		**Climate**	
Mean annual temperature (ºC)	Tann[1]	Days with precipitation ≥0.1mm	DP01
Mean temperature in spring	TSpr	Days with precipitation ≥0.1mm in spring	DP01Spr
Mean temperature in summer	TSum	Days with precipitation ≥0.1mm in summer	DP01Sum
Mean temperature in autumn	TAut	Days with precipitation ≥0.1mm in autumn	DP01Aut
Mean temperature in winter	TWin	Days with precipitation ≥0.1mm in winter	DP01Win
Mean temperature in January	TJan	Days with precipitation ≥1mm	DP1
Mean temperature in July	TJul	Days with precipitation ≥1mm in spring	DP1Spr
Annual temperature range (=TJul - TJan)	TRan	Days with precipitation ≥1mm in summer	DP1Sum
Minimum annual temperature	TnAnn	Days with precipitation ≥1mm in autumn	DP1Aut
Minimum temperature in spring	TnSpr	Days with precipitation ≥1mm in winter	DP1Win
Minimum temperature in summer	TnSum	Days with precipitation ≥10mm	DP10
Minimum temperature in autumn	TnAut	Days with precipitation ≥10mm in spring	DP10Spr
Minimum temperature in winter	TnWin	Days with precipitation ≥10mm in summer	DP10Sum
Minimum temperature in January	TnJan	Days with precipitation ≥10mm in autumn	DP10Aut
Minimum temperature in July	TnJul	Days with precipitation ≥10mm in winter	DP10Win
Maximum annual temperature	TxAnn	Days with precipitation ≥30mm	DP30
Maximum temperature in spring	TxSpr	Days with precipitation ≥30mm in spring	DP30Spr
Maximum temperature in summer	TxSum	Days with precipitation ≥30mm in summer	DP30Sum
Maximum temperature in autumn	TxAut	Days with precipitation ≥30mm in autumn	DP30Aut
Maximum temperature in winter	TxWin	Days with precipitation ≥30mm in winter	DP30Win
Maximum temperature in January	TxJan	Mean annual runoff (mm)	ROff[3]
Maximum temperature in July	TxJul	Mean annual potential evapotranspiration (mm)	PET[4]
Direct Irradiance at Surface (kWh/m^2/d)	SID[2]	Mean annual actual evapotranspiration (mm) (=min [PAnn, PET])	AET
Direct Irradiance at Surface in spring	SIDSpr	**Topography**	

Direct Irradiance at Surface in summer	SIDSum	Mean altitude (m)	Alt[5]
Direct Irradiance at Surface in autumn	SIDAut	Altitude difference (calculated from Alt)	DAlt
Direct Irradiance at Surface in winter	SIDWin	Slope (º) (calculated from Alt)	Slop
Surface Incoming Radiation (kWh/m^2/d)	SIS	Southward exposition degree	SE[6]
Surface Incoming Radiation in spring	SISSpr	Westward exposition degree	WE[6]
Surface Incoming Radiation in summer	SISSum	Hydrologically conditioned compound topographic index	CTI[7]
Surface Incoming Radiation in autumn	SISAut	**Lithology**	
Surface Incoming Radiation in winter	SISWin	Proportion of clay	Clay[8]
Days with minimum temperature ≤0ºC	DTn0	Proportion of silica	Sil
Days with minimum temperature ≤0ºC in spring	DTn0Spr	Proportion of calcareous rock	Calc
Days with minimum temperature ≤0ºC in summer	DTn0Sum	Proportion of gravels	Grav
Days with minimum temperature ≤0ºC in autumn	DTn0Aut	Proportion of gypsum	Gyps
Days with minimum temperature ≤0ºC in winter	DTn0Win	**Human activity**	
Days with minimum temperature ≥20ºC	DTn20	Distance to the nearest highway (km)	DHi[9]
Days with minimum temperature ≥20ºC in autumn	DTn20Aut	Distance to the nearest urban centre with more than 100,000 inhabitants (km)	U100
Days with maximum temperature ≥25ºC	DTx25	Distance to the nearest urban centre with more than 500,000 inhabitants (km)	U500
Days with maximum temperature ≥25ºC in spring	DTx25Spr	Human population density (number of inhabitants/km^2)	HPd[10]
Days with maximum temperature ≥25ºC in summer	DTx25Sum	**Land use**	
Days with maximum temperature ≥25ºC in autumn	DTx25Aut	Proportion of non-irrigated arable land	NIAL[11]
Total annual precipitation (mm)	PAnn	Proportion of pastures	Past
Total precipitation in spring	PSpr	Proportion of broad-leaved forest	BLF
Total precipitation in summer	PSum	Proportion of coniferous forest	CF
Total precipitation in autumn	PAut	Proportion of mixed forest	MF
Total precipitation in winter	PWin	Proportion of shrublands	Shrub

Sources and original resolutions:

[1] from *Tann* to *TxJul* and from *DTn0* to *DP30Win*: Agencia Estatal de Meteorología [1]. Original resolution: 1 km^2

[2] from *SID* to *SISWin*: Sancho et al. [2]. Original resolution: 3x3 km^2

[3] IGME [3]. Original resolution: ~1 km^2

[4] from *PET* to *AET*: Font [4]. Original resolution: ~1 km^2

[5] US Geological Survey [5]. Original resolution: ~1 km^2

[6] Farr and Kobrick [6]. Original resolution: ~100x100 m^2

[7] http://hydrosheds.cr.usgs.gov. Original resolution: ~600x600 m^2

[8] from *Clay* to *Gyps*: OneGeology-Europe [7]. Original resolution: ~100x100 m^2

[9] from *DHi* to *U500*: IGN [8]. Original resolution: ~1 km^2
[10] ORNL [9]. Original resolution: ~1 km^2
[11] from *NIAL* to *Shrub*: CLC [10]. Original data in vector format.

References

1. Agencia Estatal de Meteorología. Iberian climate atlas. Air temperature and precipitation (1971-2000): Agencia Estatal de Meteorología. Ministerio de Medio Ambiente y Medio Rural y Marino; 2011.
2. Sancho JM, Riesco J, Jiménez C, Sánchez de Cos MC, Montero J, López M. Atlas de radiación solar en España utilizando datos del SAF de clima de EUMETSAT: Agencia Estatal de Meteorología. Ministerio de Agricultura, Alimentación y Medio Ambiente; 2012.
3. IGME. Mapa hidrogeológico nacional. Explicación de los mapas de lluvia útil, de reconocimiento hidrogeológico y de síntesis de los sistemas acuíferos. Vol. 81, 2ª ed. Madrid: Instituto Geológico y Minero de España. Ministerio de Industria y Energía; 1979.
4. Font I. Climatología de España y Portugal: Ediciones Universidad de Salamanca; 2000.
5. US Geological Survey. GTOPO30. Land Processes Distributed Active Archive Center. <http://www.edcdaac.usgs.gov/gtopo30/gtopo30.asp>. 1996.
6. Farr TG, Kobrick M. Shuttle radar topography mission produces a wealth of data. Eos Trans Am Geophys Union. 2000; 81: 583–585.
7. OneGeology-Europe. http://www.onegeology-europe.org/. 2015.
8. IGN. Mapa de carreteras. Península Ibérica, Baleares y Canarias. Madrid: Instituto Geográfico Nacional, Ministerio de Fomento; 1999.
9. ORNL. LandScan 2000 Global Population Database. Oak Ridge, TN: Oak Ridge National Laboratory (ORNL); 2001.
10. CLC. CORINE Land Cover 2000. Downloaded from http://centrodedescargas.cnig.es/CentroDescargas/index.jsp; 2000.
