# Supplementary material for: Assessment of the National Park network of mainland Spain by the Insecurity Index of vertebrate species
Source: PLoS One. 2018 May 21;13(5):e0197496. doi: 10.1371/journal.pone.0197496 (PMC5962089; doi:10.1371/journal.pone.0197496)
Supplement: S2 Table — (PDF) [file pone.0197496.s002.pdf]

Assessment of the National Park network of mainland Spain by the Insecurity Index of vertebrate species

Alba Estrada & Raimundo Real

Plos One

S2 Table. Representativeness of observed species occurrence in the National Park network.

Species	Representativeness
Chioglossa lusitanica	0.900
Euproctus asper	4.198
Pleurodeles waltl	1.499
Bufo calamita	0.900
Hyla meridionalis	1.349
Rana iberica	1.349
Rana pyrenaica	17.691
Emys orbicularis	2.249
Mauremys leprosa	1.349
Testudo graeca	18.291
Anguis fragilis	1.649
Podarcis muralis	3.598
Elaphe scalaris	1.049
Phalacrocorax aristotelis	3.448
Ciconia nigra	1.799
Netta rufina	5.097
Gypaetus barbatus	3.748
Neophron percnopterus	1.349
Aegypus monachus	8.546
Aquila adalberti	10.795
Aquila chrysaetos	2.399
Lagopus mutus	10.795
Tetrao urogallus	5.847
Perdix perdix	6.447
Otis tarda	0.150
Picus viridis	0.900
Pyrrhocorax graculus	7.046
Pyrrhocorax pyrrhocorax	1.949
Galemys pyrenaicus	3.148
Canis lupus	1.049
Lutra lutra	1.199
Ursus arctos	3.748
Felis silvestris	2.249
Lynx pardinus	16.942
Cervus elaphus	1.949
Rupicapra pyrenaica	7.946
Capra pyrenaica	2.849
