# Supplementary figures and images for: Assessment of the National Park network of mainland Spain by the Insecurity Index of vertebrate species
Source: PLoS One. 2018 May 21;13(5):e0197496. doi: 10.1371/journal.pone.0197496 (PMC5962089; doi:10.1371/journal.pone.0197496)

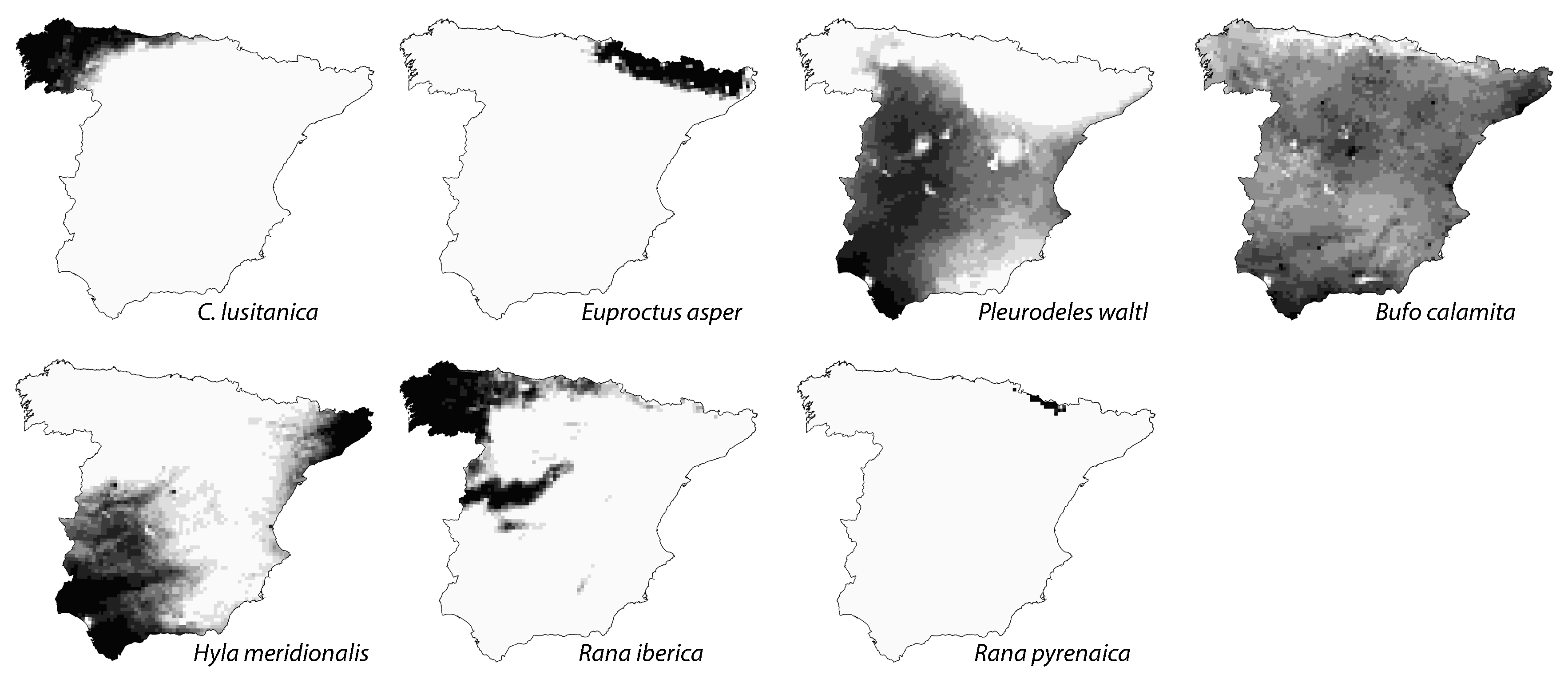

Supplement: S1 Fig — Values range from zero (white cells) to one (black cells). (TIF) [file pone.0197496.s003.tif]

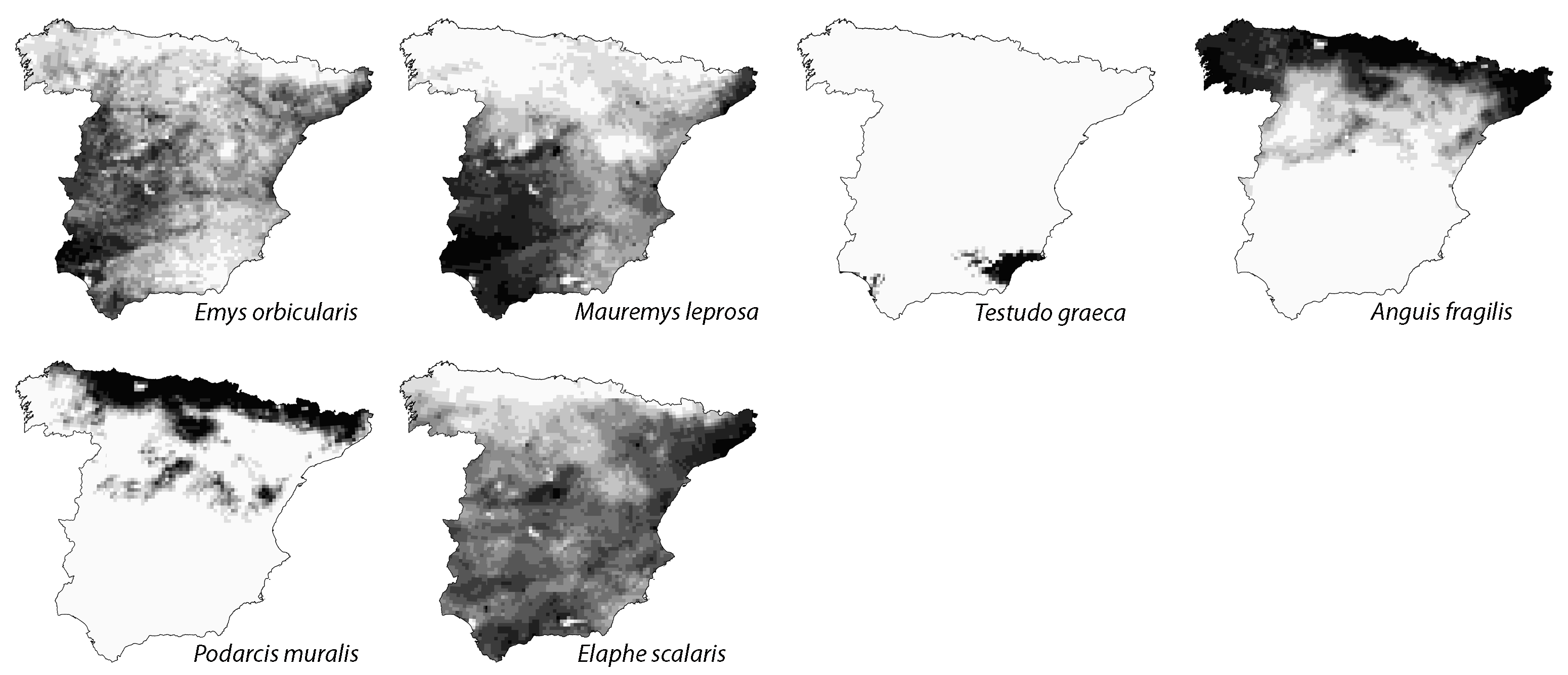

Supplement: S2 Fig — Values range from zero (white cells) to one (black cells). (TIF) [file pone.0197496.s004.tif]

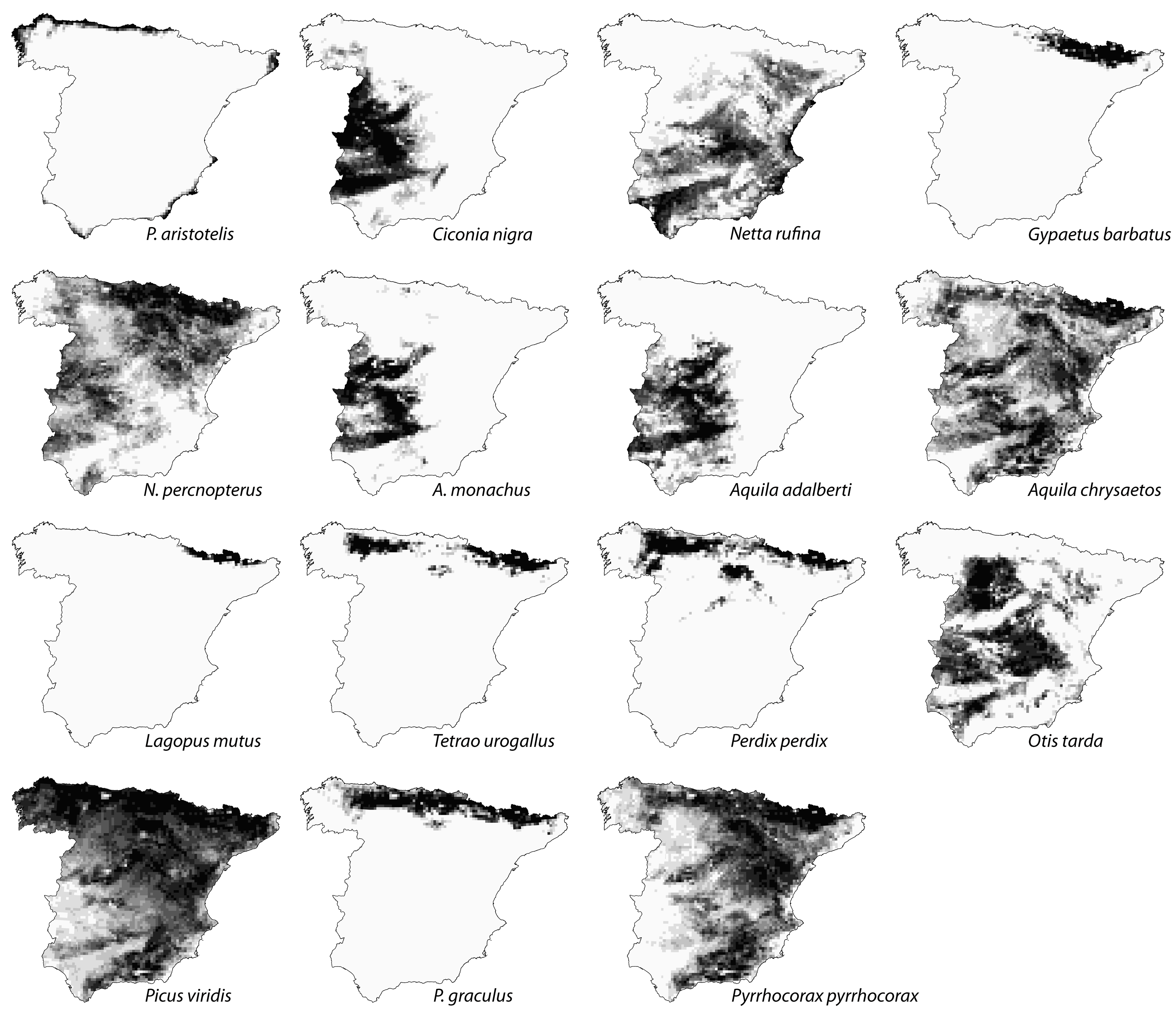

Supplement: S3 Fig — Values range from zero (white cells) to one (black cells). (TIF) [file pone.0197496.s005.tif]

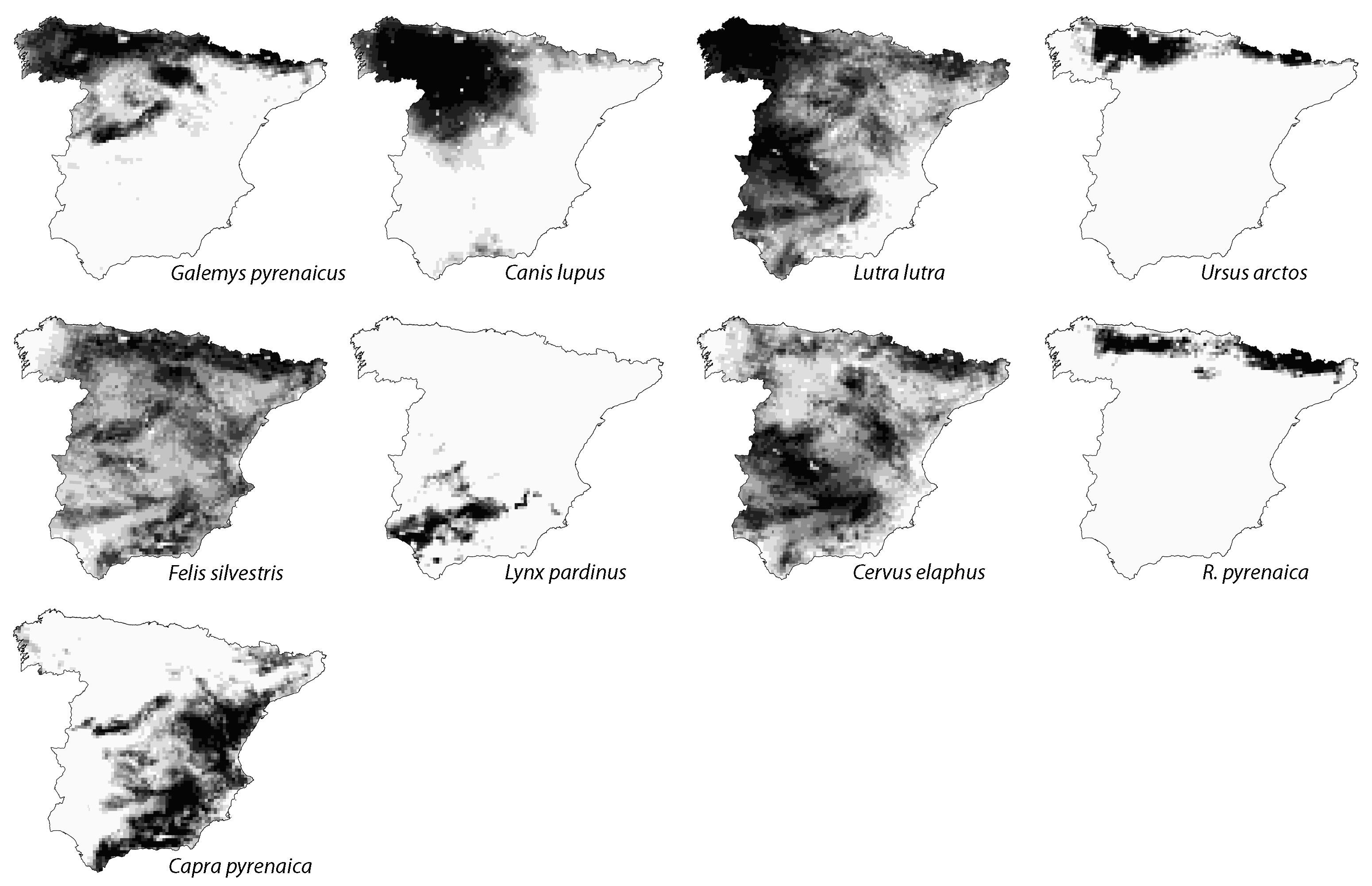

Supplement: S4 Fig — Values range from zero (white cells) to one (black cells). (TIF) [file pone.0197496.s006.tif]
