# Supplementary material for: Assessment of the National Park network of mainland Spain by the Insecurity Index of vertebrate species
Source: PLoS One. 2018 May 21;13(5):e0197496. doi: 10.1371/journal.pone.0197496 (PMC5962089; doi:10.1371/journal.pone.0197496)
Supplement: S1 Appendix — (PDF) [file pone.0197496.s007.pdf]

Assessment of the National Park network of mainland Spain by the Insecurity Index of vertebrate species

Alba Estrada & Raimundo Real

Plos One

S1 Appendix. Variables included at each step of the modelling process and final favourability models. Variables included at each step of the modelling process (i.e., pairwise correlations, spatial descriptor, FDR, and forward-backward stepwise) for each species. X: longitude; Y: latitude. Variables selected for the spatial descriptor were those selected in the trend surface approach (spatial model). The variables included in the forward-backward stepwise are those of the final favourability models. Variables are sorted according to their order of entrance in the models. β: coefficients; SE: standard errors; Sig: significance: ***<0.001, **<0.01, *<0.05, ns>0.05. Evaluation metrics of the models are also shown. Variable codes as in S1 Table. "Spatial" in final favourability models is the linear combination of selected spatial variables with their coefficients and the intercept of the spatial model (see Estrada et al. 2016 Plos One).

Chioglossa lusitanica

Pairwise correlations, spatial descriptor and FDR

Human, lithology and land use variables were not correlated, thus all of them were selected in pairwise correlations.

Variables selected			
**Climate**	**Topography**	**Spatial**	**Variables excluded by the FDR**
DP1Win	Alt	XY	HPd
DP30Spr	SE	X^2	DTn0Sum
DP30Sum	WE	X^2Y	Shrub
DTn0Win	CTI	XY^2	
DTn0Sum			
DTn20			
DTx25Spr			
TRan			
SIDAut			
AET			

Final favourability model

Variables	ß	SE	Sig
(Intercept)	14.104053	4.332457	**
Spatial	0.75198	0.196765	***
Sil	4.340705	0.923729	***
DP30Spr	0.236318	0.135775	ns
DTn0Win	-0.042055	0.010895	***
CTI	-0.906338	0.251222	***
DHi	-0.015947	0.007593	*
DTn20	-0.435574	0.193528	*
Clay	3.87317	1.439383	**
Calc	2.86569	1.209104	*
BLF	-1.552821	0.887971	ns
Grav	4.034756	2.281961	ns
SE	-0.016587	0.010997	ns

Evaluation metrics	
AUC	0.9853935
Kappa	0.4657085
Sensitivity	0.9880952
Specificity	0.9334630
CCR	0.935192
HL	11.3658311 ns

Euproctus asper

Pairwise correlations, spatial descriptor and FDR

Human, lithology and land use variables were not correlated, thus all of them were selected in pairwise correlations.

Variables selected

Climate	Topography	Spatial
DP10Spr	Alt	X
DP10Sum	SE	XY
DTn0Win	WE	X^2
DTn0Sum	CTI	X^2Y
DTn20		XY^2
TRan		
AET		

Variables excluded by the FDR
Shrub
WE
Gyps

Final favourability model

Variables	ß	SE	Sig
(Intercept)	-2.965059	2.667609	ns
Spatial	1.481486	0.222381	***
BLF	3.721701	0.98703	***
HPd	-0.024717	0.014962	ns
DTn20	-0.189029	0.045073	***
U500	-0.018825	0.007658	*
NIAL	-7.78196	2.232869	***
Past	-7.26826	2.898036	*
MF	2.914323	1.988447	ns
Alt	0.003819	0.001015	***
DTn0Win	-0.105248	0.028221	***
TRan	0.542185	0.189932	**
DTn0Sum	-0.625408	0.407322	ns

Evaluation metrics	
AUC	0.9960155
Kappa	0.7169263
Sensitivity	1
Specificity	0.9726509
CCR	0.9736247
HL	22.6335351 **

Pleurodeles waltl

Pairwise correlations, spatial descriptor and FDR

Human, lithology and land use variables were not correlated, thus all of them were selected in pairwise correlations.

Variables selected			
**Climate**	**Topography**	**Spatial**	**Variables excluded by the FDR**
DP10Spr	Alt	Y	Shrub
DTn0Sum	DAlt	X	HPd
DTn20	SE	XY	SE
TRan	WE	X^2	BLF
TxAut	CTI	X^3	Clay
SIDSum		Y^2	
SISWin		Y^3	
AET		X^2Y	
		XY^2	

Final favourability model

Variables	ß	SE	Sig
(Intercept)	3.97E+00	1.45E+00	**
Spatial	1.06E+00	6.04E-02	***
DTn0Sum	-4.15E+00	6.50E-01	***
Alt	6.67E-04	1.59E-04	***
U500	2.44E-03	7.90E-04	**
Gyps	-1.52E+01	1.13E+01	ns
CTI	-1.59E-01	6.07E-02	**
Grav	6.01E-01	2.33E-01	**
DP10Spr	-1.39E-01	4.20E-02	***
AET	8.82E-04	4.52E-04	ns
Sil	2.73E-01	1.31E-01	*
SIDSum	-2.94E-01	1.53E-01	ns

Evaluation metrics	
AUC	0.8575984
Kappa	0.4343372
Sensitivity	0.8536386
Specificity	0.7072215
CCR	0.7409570
HL	50.1243829 ***

Bufo calamita

Pairwise correlations, spatial descriptor and FDR

Human, lithology and land use variables were not correlated, thus all of them were selected in pairwise correlations.

Variables selected			
**Climate**	**Topography**	**Spatial**	**Variables excluded by the FDR**
DP1	Alt	Y	DTx25Spr
DP30Aut	Slop	X	WE
DP30Sum	SE	XY	Shrub
DTn0Sum	WE	X^2	DHi
DTn20		X^3	Calc
DTx25Spr		Y^2	Gyps
TRan		Y^3	Alt
TnJul		XY^2	
AET			

Final favourability model

Variables	ß	SE	Sig
(Intercept)	1.12E+00	4.17E-01	**
Spatial	7.69E-01	7.40E-02	***
U100	-4.69E-03	1.13E-03	***
Past	-5.61E+00	9.39E-01	***
HPd	8.78E-04	2.05E-04	***
CF	1.10E+00	2.44E-01	***
BLF	7.82E-01	2.79E-01	**
DP30Aut	-3.18E-01	4.30E-02	***
Sil	5.15E-01	8.99E-02	***
U500	-3.52E-03	6.07E-04	***
TRan	-5.64E-02	1.81E-02	**
AET	7.33E-04	2.90E-04	*
SE	0.0034787	0.0022651	ns

Evaluation metrics	
AUC	0.6886401
Kappa	0.2716559
Sensitivity	0.6611627
Specificity	0.6109954
CCR	0.6384702
HL	40.4083661 ***

Hyla meridionalis

Pairwise correlations, spatial descriptor and FDR

Human, lithology and land use variables were not correlated, thus all of them were selected in pairwise correlations.

Variables selected			
**Climate**	**Topography**	**Spatial**	**Variables excluded by the FDR**
DP30Win	Alt	Y	CF
DP30Sum	DAlt	XY	Gyps
DTn0Sum	SE	X^3	TRan
DTn20	WE	Y^2	Shrub
TAut	CTI	Y^3	DHi
TRan		X^2Y	
ROff		XY^2	
AET			

Final favourability model

Variables	ß	SE	Sig
(Intercept)	1.91E+00	1.99E+00	ns
Spatial	8.52E-01	4.14E-02	***
AET	2.52E-03	5.07E-04	***
DTn20	1.85E-02	4.88E-03	***
Sil	-1.16E+00	4.46E-01	**
SE	1.59E-02	4.68E-03	***
Alt	-1.09E-03	2.80E-04	***
CTI	-2.40E-01	8.81E-02	**
HPd	3.84E-04	1.35E-04	**
Past	5.48E+00	2.15E+00	*
Clay	-2.06E+00	4.67E-01	***
WE	8.26E-03	5.75E-03	ns
BLF	1.2950793	0.6347605	*
Grav	-1.9231928	0.5357702	***
Calc	-1.7108759	0.4807249	***

Evaluation metrics	
AUC	0.9494523
Kappa	0.5852454
Sensitivity	0.9322251
Specificity	0.8486522
CCR	0.8609646
HL	56.7003716 ***

Rana iberica

Pairwise correlations, spatial descriptor and FDR

Human, lithology and land use variables were not correlated, thus all of them were selected in pairwise correlations.

Variables selected			
**Climate**	**Topography**	**Spatial**	**Variables excluded by the FDR**
DP10Win	Alt	Y	Alt
DP30Spr	DAlt	X	SE
DP30Sum	SE	XY	HPd
DTn0Sum	WE	X^2	
DTn20	CTI	X^3	
TRan		Y^2	
SISWin		Y^3	
PET		X^2Y	
AET		XY^2	

Final favourability model

Variables	ß	SE	Sig
(Intercept)	-6.66E+00	1.80E+00	***
Spatial	1.04E+00	1.08E-01	***
DAlt	1.20E-03	3.16E-04	***
Sil	5.92E+00	8.51E-01	***
U500	-9.78E-03	1.72E-03	***
CF	4.13E+00	8.44E-01	***
PET	-1.75E-02	2.99E-03	***
AET	1.08E-02	2.29E-03	***
DP30Sum	-1.33E+00	3.23E-01	***
Calc	5.80E+00	9.66E-01	***
Grav	6.10E+00	1.19E+00	***
DP10Win	1.09E-01	3.31E-02	**
Clay	3.7404198	1.0882839	***
SISWin	4.8799604	0.983512	***
TRan	-0.2966148	0.0723339	***
DTn0Sum	0.5978934	0.259484	*
MF	2.5633932	0.8419069	**
BLF	2.1377256	0.7343627	**
Shrub	1.2962815	0.5504615	*

Evaluation metrics	
AUC	0.9834648
Kappa	0.6569974
Sensitivity	0.9522822
Specificity	0.9218815
CCR	0.9246420
HL	20.9191192 **

Rana pyrenaica

Pairwise correlations, spatial descriptor and FDR

Human, lithology and land use variables were not correlated, thus all of them were selected in pairwise correlations.

Variables selected			
**Climate**	**Topography**	**Spatial**	**Variables excluded by the FDR**
DP10Spr	Alt	X	WE
DP10Sum	SE	XY	Gyps
DTn0Sum	WE	X^3	BLF
DTn20	CTI	Y^2	Past
TRan		Y^3	Sil
TxAut		X^2Y	U500
AET		XY^2	

Final favourability model

Variables	ß	SE	Sig
(Intercept)	3.31E+02	1.09E+02	**
Spatial	4.05E+01	1.29E+01	**
Calc	1.23E+02	3.98E+01	**
CF	4.07E+02	1.31E+02	**
Shrub	-2.49E+02	8.74E+01	**
Clay	3.68E+02	1.19E+02	**
DHi	-1.78E+00	6.93E-01	*
TRan	-2.24E+01	7.59E+00	**
MF	4.22E+01	1.84E+01	*

Evaluation metrics	
AUC	1
Kappa	0.9051913
Sensitivity	1
Specificity	0.9990537
CCR	0.9990580
HL	0.9031554 ns

Emys orbicularis

Pairwise correlations, spatial descriptor and FDR

Human, lithology and land use variables were not correlated, thus all of them were selected in pairwise correlations.

Variables selected			
**Climate**	**Topography**	**Spatial**	**Variables excluded by the FDR**
DP10Sum	Alt	Y	Grav
DP30Spr	DAlt	X	Gyps
DTn0Sum	SE	XY	U100
DTn20	WE	X^3	WE
TRan	CTI	Y^2	
TxAut		Y^3	
ROff		X^2Y	
AET		XY^2	

Final favourability model

Variables	ß	SE	Sig
(Intercept)	-3.98E-01	2.08E+00	ns
Spatial	5.16E-01	7.71E-02	***
NIAL	-6.23E-01	3.10E-01	*
DTn0Sum	-1.75E+00	6.98E-01	*
Calc	-8.82E-01	2.20E-01	***
DHi	1.49E-02	2.70E-03	***
U500	-4.85E-03	1.07E-03	***
BLF	2.61E+00	4.85E-01	***
DP10Sum	-3.56E-01	7.44E-02	***
Clay	-5.80E-01	2.44E-01	*
SE	1.22E-02	4.37E-03	**
CTI	2.44E-01	6.91E-02	***
Shrub	1.1467561	0.3312632	***
TxAut	-0.2690325	0.0569823	***
Alt	-0.0017543	0.0003831	***
TRan	0.0802111	0.033057	*
CF	1.1775007	0.5300833	*

Evaluation metrics	
AUC	0.8112590
Kappa	0.2116908
Sensitivity	0.7651007
Specificity	0.7183707
CCR	0.7223060
HL	20.8598058 **

Mauremys leprosa

Pairwise correlations, spatial descriptor and FDR

Human, lithology and land use variables were not correlated, thus all of them were selected in pairwise correlations.

Variables selected			
**Climate**	**Topography**	**Spatial**	**Variables excluded by the FDR**
DP30Sum	Alt	X	Clay
DTn0Spr	DAlt	XY	Grav
DTn0Sum	SE	X^3	U100
DTn20	WE	Y^2	
PSpr	CTI	Y^3	
TJul		X^2Y	
TRan			
AET			

Final favourability model

Variables	ß	SE	Sig
(Intercept)	-9.14E-01	4.15E-01	*
Spatial	6.38E-01	4.25E-02	***
DTn0Spr	-4.82E-02	7.65E-03	***
Sil	5.55E-01	1.23E-01	***
U500	-5.59E-03	7.78E-04	***
DTn0Sum	-2.75E+00	6.57E-01	***
Shrub	8.07E-01	2.27E-01	***
HPd	5.89E-04	1.40E-04	***
CF	1.24E+00	3.52E-01	***
PSpr	3.26E-03	9.92E-04	***
DP30Sum	-4.32E-01	1.34E-01	**
Past	-3.68E+00	1.77E+00	*
DHi	0.0039865	0.001976	*
BLF	0.8579009	0.4324115	*
TRan	0.0558105	0.022311	*
Gyps	-0.8969753	0.5267162	ns
NIAL	-0.3628105	0.2004525	ns

Evaluation metrics	
AUC	0.8572861
Kappa	0.5183574
Sensitivity	0.8115038
Specificity	0.7743855
CCR	0.7844763
HL	41.2214238 ***

Testudo graeca

Pairwise correlations, spatial descriptor and FDR

Human, lithology and land use variables were not correlated, thus all of them were selected in pairwise correlations.

Variables selected			
**Climate**	**Topography**	**Spatial**	**Variables excluded by the FDR**
DP1	Alt	Y	Gyps
DP30Spr	SE	XY	CTI
DTn0Sum	WE	X^2	Shrub
DTn20	CTI	Y^2	CF
DTx25Aut		Y^3	Sil
PSum		X^2Y	Clay
TRan		XY^2	HPd
TxWin			U100
AET			

Final favourability model

Variables	ß	SE	Sig
(Intercept)	5.87E+00	3.57E+00	ns
Spatial	1.00E+00	2.15E-01	***
BLF	-1.92E+02	1.14E+02	ns
WE	-5.91E-02	2.29E-02	**
NIAL	-6.81E+00	5.18E+00	ns
DTn20	-2.80E-02	1.32E-02	*
SE	-4.06E-02	1.86E-02	*
DTx25Aut	1.14E-01	5.60E-02	*

Evaluation metrics	
AUC	0.9981490
Kappa	0.4687089
Sensitivity	1
Specificity	0.9844432
CCR	0.9845516
HL	11.5754054 ns

Anguis fragilis

Pairwise correlations, spatial descriptor and FDR

Human, lithology and land use variables were not correlated, thus all of them were selected in pairwise correlations.

Variables selected			
**Climate**	**Topography**	**Spatial**	**Variables excluded by the FDR**
DP01	Alt	Y	SE
DP30Spr	SE	X	Calc
DP30Sum	WE	X^2	WE
DTn0Sum	CTI	X^3	
DTn20		Y^2	
TRan		Y^3	
TxJul		X^2Y	
PET		XY^2	
AET			

Final favourability model

Variables	ß	SE	Sig
(Intercept)	2.67E+00	1.11E+00	*
Spatial	6.08E-01	4.72E-02	***
AET	4.05E-03	6.73E-04	***
DP30Sum	5.89E-01	1.49E-01	***
DHi	-1.54E-02	3.18E-03	***
PET	-5.92E-03	1.30E-03	***
U500	-4.85E-03	1.11E-03	***
HPd	3.51E-04	1.36E-04	**
BLF	1.47E+00	3.92E-01	***
CF	1.63E+00	4.18E-01	***
Past	-1.67E+00	6.57E-01	*
TRan	-6.15E-02	2.91E-02	*
DTn20	0.0203212	0.0081187	*
U100	-0.0040251	0.0024434	ns
DTn0Sum	0.2843962	0.1636721	ns
Sil	0.4647558	0.1717781	**
Clay	0.4879699	0.2540485	ns

Evaluation metrics	
AUC	0.9391049
Kappa	0.6307209
Sensitivity	0.9068136
Specificity	0.8540603
CCR	0.8639789
HL	11.6840720 ns

Podarcis muralis

Pairwise correlations, spatial descriptor and FDR

Human, lithology and land use variables were not correlated, thus all of them were selected in pairwise correlations.

Variables selected			
**Climate**	**Topography**	**Spatial**	**Variables excluded by the FDR**
DP01Sum	Alt	Y	U500
DP30Sum	SE	X	WE
DTn0Sum	WE	XY	DHi
DTn20	CTI	X^2	HPd
PSpr		X^3	
TRan		Y^2	
TxInv		Y^3	
AET		X^2Y	
		XY^2	

Final favourability model

Variables	ß	SE	Sig
(Intercept)	-7.03E+00	1.63E+00	***
Spatial	7.69E-01	5.21E-02	***
DTn20	-5.60E-02	1.77E-02	**
CF	3.47E+00	5.57E-01	***
U100	-2.85E-02	3.39E-03	***
Alt	3.86E-03	5.44E-04	***
BLF	2.74E+00	5.48E-01	***
AET	5.97E-03	1.33E-03	***
TxWin	2.75E-01	9.01E-02	**
SE	-2.64E-02	6.88E-03	***
DP30Sum	6.32E-01	1.95E-01	**
Sil	5.14E-01	2.11E-01	*
NIAL	-1.1542659	0.5112433	*

Evaluation metrics	
AUC	0.9790950
Kappa	0.6890698
Sensitivity	0.9391447
Specificity	0.9191489
CCR	0.921439
HL	17.4712761 *

Elaphe scalaris

Pairwise correlations, spatial descriptor and FDR

Human, lithology and land use variables were not correlated, thus all of them were selected in pairwise correlations.

Variables selected			
**Climate**	**Topography**	**Spatial**	**Variables excluded by the FDR**
DP30Spr	Alt	Y	WE
DP30Sum	SE	X	SE
DTn0Spr	WE	XY	Calc
DTn0Sum	CTI	X^2	NIAL
DTn20		X^3	Clay
DTx25Spr		Y^2	
DTx25Sum		Y^3	
TRan		X^2Y	
ROff		XY^2	
AET			

Final favourability model

Variables	ß	SE	Sig
(Intercept)	1.62E+00	8.56E-01	ns
Spatial	6.78E-01	6.43E-02	***
DTn0Spr	-4.97E-02	5.73E-03	***
DTn20	-1.16E-02	3.16E-03	***
CTI	-2.74E-01	4.37E-02	***
HPd	5.90E-04	1.75E-04	***
DTx25Sum	2.71E-02	5.91E-03	***
DTx25Spr	-5.48E-02	7.23E-03	***
U100	-7.62E-03	1.26E-03	***
TRan	1.47E-01	2.62E-02	***
AET	9.25E-04	2.74E-04	***
Grav	7.58E-01	1.88E-01	***
Sil	0.3484844	0.0993564	***
U500	-0.0019736	0.0006779	**
Past	-3.0309997	1.3219593	*
DTn0Sum	0.4530161	0.2208598	*
CF	0.4398373	0.2779301	ns

Evaluation metrics	
AUC	0.7409146
Kappa	0.3028314
Sensitivity	0.7317588
Specificity	0.5784814
CCR	0.6469480
HL	73.9301215 ***

Phalacrocorax aristotelis

Pairwise correlations, spatial descriptor and FDR

Human, lithology and land use variables were not correlated, thus all of them were selected in pairwise correlations.

Variables selected			
**Climate**	**Topography**	**Spatial**	**Variables excluded by the FDR**
DP30Sum	Alt	Y	Calc
DTn0Win	DAlt	X	WE
DTn0Sum	SE	XY	Shrub
DTn20Aut	WE	X^2	
DTx25Spr	CTI	X^3	
DTx25Sum		Y^2	
PAut		Y^3	
TRan		X^2Y	
AET		XY^2	

Final favourability model

Variables	ß	SE	Sig
(Intercept)	2.99E+01	5.54E+00	***
Spatial	5.01E-01	1.15E-01	***
DTn0Sum	-2.57E+01	1.55E+01	ns
CTI	-1.14E+00	2.95E-01	***
Alt	-9.30E-03	2.78E-03	***
SE	-2.11E-02	1.41E-02	ns
U100	5.73E-02	1.27E-02	***
MF	-5.01E+00	1.40E+00	***
TRan	-6.81E-01	1.62E-01	***
BLF	-5.14E+00	1.43E+00	***
DHi	-4.19E-02	1.67E-02	*

Evaluation metrics	
AUC	0.9949604
Kappa	0.4715157
Sensitivity	0.9893617
Specificity	0.9624089
CCR	0.9628862
HL	13.5115766 ns

Ciconia nigra

Pairwise correlations, spatial descriptor and FDR

Human, lithology and land use variables were not correlated, thus all of them were selected in pairwise correlations.

Variables selected			
**Climate**	**Topography**	**Spatial**	**Variables excluded by the FDR**
DP10Win	Alt	Y	SE
DP30Spr	DAlt	X	CTI
DP30Sum	SE	XY	
DTn0Spr	WE	X^2	
DTn0Sum	CTI	X^3	
DTn20		X^2Y	
DTx25Spr		XY^2	
TRan			
SIDWin			
SIDSum			
AET			

Final favourability model

Variables	ß	SE	Sig
(Intercept)	-2.25E+00	1.73E+00	ns
Spatial	6.52E-01	7.58E-02	***
AET	3.92E-03	6.99E-04	***
HPd	-1.65E-02	7.89E-03	*
NIAL	-2.50E+00	5.66E-01	***
DTn0Sum	-1.71E+00	1.06E+00	ns
DHi	1.71E-02	3.29E-03	***
TRan	3.21E-01	6.73E-02	***
Sil	7.39E-01	2.72E-01	**
DP30Sum	-2.33E+00	6.52E-01	***
CF	2.86E+00	7.61E-01	***
Past	-3.98E+00	2.82E+00	ns
SIDWin	-3.3990985	0.6744347	***
U500	-0.0063829	0.0017097	***
DTn0Spr	-0.0340839	0.0221235	ns
BLF	1.6387195	0.6574473	*
Alt	-0.0009056	0.0005386	ns
Grav	-0.9810976	0.6603876	ns

Evaluation metrics	
AUC	0.9619832
Kappa	0.5469590
Sensitivity	0.9539749
Specificity	0.8801242
CCR	0.8867747
HL	7.8993221 ns

Netta rufina

Pairwise correlations, spatial descriptor and FDR

Human, lithology and land use variables were not correlated, thus all of them were selected in pairwise correlations.

Variables selected			
**Climate**	**Topography**	**Spatial**	**Variables excluded by the FDR**
DP30Sum	Alt	XY	Clay
DTn0Sum	DAlt	X^2	NIAL
DTn20	SE	X^3	HPd
TRan	WE	Y^2	TRan
SIDSpr	CTI	Y^3	Gyps
AET		X^2Y	SE
		XY^2	WE

Final favourability model

Variables	ß	SE	Sig
(Intercept)	-1.73E+01	2.54E+00	***
CTI	5.94E-01	1.03E-01	***
Spatial	4.89E-01	9.27E-02	***
SIDSpr	1.24E+00	5.06E-01	*
MF	-1.99E+01	1.50E+01	ns
Grav	7.86E-01	3.83E-01	*
CF	-3.69E+00	1.53E+00	*
BLF	-4.74E+00	2.71E+00	ns
Sil	-1.18E+00	4.70E-01	*
DHi	1.33E-02	5.48E-03	*
DTn20	9.35E-03	5.89E-03	ns

Evaluation metrics	
AUC	0.9130220
Kappa	0.1535811
Sensitivity	0.9113924
Specificity	0.7768932
CCR	0.7808968
HL	11.3447743 ns

Gypaetus barbatus

Pairwise correlations, spatial descriptor and FDR

Human, lithology and land use variables were not correlated, thus all of them were selected in pairwise correlations.

Variables selected			
**Climate**	**Topography**	**Spatial**	**Variables excluded by the FDR**
DP10Spr	Alt	X	Sil
DP10Sum	DAlt	X^3	WE
DTn0Win	SE	Y^2	U500
DTn0Sum	WE	Y^3	Gyps
DTn20Aut	CTI	XY^2	TRan
TRan			
AET			

Final favourability model

Variables	ß	SE	Sig
(Intercept)	4.09E+01	1.25E+01	**
Spatial	1.20E+00	1.87E-01	***
DAlt	3.65E-03	7.99E-04	***
U100	-2.45E-02	1.02E-02	*
Shrub	5.17E+00	1.61E+00	**
CTI	-2.62E+00	7.10E-01	***
DP10Sum	-4.18E-01	1.96E-01	*
SE	3.98E-02	1.95E-02	*
MF	4.22E+00	1.84E+00	*
CF	-2.00E+00	1.27E+00	ns
DTn0Win	-3.14E-02	1.34E-02	*

Evaluation metrics	
AUC	0.9952292
Kappa	0.5098472
Sensitivity	1
Specificity	0.9687680
CCR	0.9692916
HL	9.6854071 ns

Neophron percnopterus

Pairwise correlations, spatial descriptor and FDR

Human, lithology and land use variables were not correlated, thus all of them were selected in pairwise correlations.

Variables selected			
**Climate**	**Topography**	**Spatial**	**Variables excluded by the FDR**
DP10Sum	Alt	XY	WE
DP30Spr	DAlt	X^2	TRan
DTn0Sum	SE	X^3	U500
DTn20	WE	Y^2	Sil
TRan	CTI	Y^3	
TxWin		X^2Y	
SISWin		XY^2	
ROff			
AET			

Final favourability model

Variables	ß	SE	Sig
(Intercept)	6.94E+00	1.96E+00	***
Spatial	1.16E+00	6.99E-02	***
Shrub	2.45E+00	2.76E-01	***
DHi	1.54E-02	2.11E-03	***
BLF	1.78E+00	3.77E-01	***
DTn20	1.25E-02	5.42E-03	*
Calc	9.70E-01	1.44E-01	***
U100	1.08E-02	1.70E-03	***
MF	2.36E+00	5.70E-01	***
SE	-1.72E-02	3.63E-03	***
DAlt	1.30E-03	2.43E-04	***
DP10Sum	-2.12E-01	4.63E-02	***
Alt	-0.0021521	0.0002819	***
TxWin	-0.2564922	0.0442536	***
CTI	-0.2198655	0.095337	*
DP30Spr	-0.2524642	0.0885915	**
NIAL	-0.679976	0.271057	*
Clay	0.429391	0.1893503	*
AET	0.0009067	0.0004482	*
Gyps	0.6010296	0.3554959	ns

Evaluation metrics	
AUC	0.8543394
Kappa	0.3870012
Sensitivity	0.7877155
Specificity	0.7481735
CCR	0.7550867
HL	72.8182265 ***

Aegypus monachus

Pairwise correlations, spatial descriptor and FDR

Human, lithology and land use variables were not correlated, thus all of them were selected in pairwise correlations.

Variables selected			
**Climate**	**Topography**	**Spatial**	**Variables excluded by the FDR**
DP30Spr	Alt	X	DAlt
DP30Sum	DAlt	XY	CF
DTn0Sum	SE	X^2	WE
DTn20	WE	X^3	DP30Spr
DTx25Spr	CTI	Y^2	Alt
TRan		Y^3	SE
TnJul		XY^2	
SIDWin			
SIDSum			
AET			

Final favourability model

Variables	ß	SE	Sig
(Intercept)	6.12E+00	4.42E+00	ns
Spatial	1.08E+00	1.64E-01	***
Shrub	2.55E+00	6.09E-01	***
BLF	3.47E+00	9.23E-01	***
DHi	2.12E-02	4.62E-03	***
NIAL	-2.03E+00	1.22E+00	ns
DTx25Spr	6.49E-02	2.00E-02	**
SIDSum	-2.95E+00	7.08E-01	***
U100	-1.18E-02	3.82E-03	**
Past	-1.25E+01	6.61E+00	ns
HPd	-2.20E-02	1.88E-02	ns
AET	4.00E-03	1.20E-03	***
TRan	0.373362	0.11373	**
Calc	-2.011601	0.92926	*
Grav	-2.374854	1.579367	ns
DTn20	-0.017868	0.012335	ns

Evaluation metrics	
AUC	0.9668476
Kappa	0.2833218
Sensitivity	0.9615385
Specificity	0.8765528
CCR	0.8790505
HL	8.0547909 ns

Aquila adalberti

Pairwise correlations, spatial descriptor and FDR

Human, lithology and land use variables were not correlated, thus all of them were selected in pairwise correlations.

Variables selected			
**Climate**	**Topography**	**Spatial**	**Variables excluded by the FDR**
DP30Spr	Alt	Y	Slop
DP30Sum	Slop	X	HPd
DTn0Sum	SE	XY	Alt
DTn20	WE	X^2	SE
DTx25Spr		X^3	CF
TRan		Y^2	U500
TnJul		Y^3	WE
SIDWin		X^2Y	
SIDSum		XY^2	
AET			

Final favourability model

Variables	ß	SE	Sig
(Intercept)	1.48E+01	5.08E+00	**
Spatial	1.06E+00	1.31E-01	***
NIAL	-3.66E+00	8.89E-01	***
Shrub	1.49E+00	5.45E-01	**
DP30Spr	-1.27E+00	2.83E-01	***
BLF	3.37E+00	9.13E-01	***
DHi	-7.26E-03	4.36E-03	ns
AET	4.47E-03	1.13E-03	***
Calc	-1.97E+00	6.75E-01	**
Clay	-1.40E+00	5.11E-01	**
TRan	2.50E-01	8.61E-02	**
SIDSum	-4.73E+00	1.03E+00	***
SIDWin	1.75943	1.003337	ns
Past	-18.897838	11.427461	ns
Grav	1.205273	0.555369	*
DTx25Spr	0.041297	0.020827	*
DTn20	-0.030661	0.014351	*
TnJul	0.154892	0.098609	ns

Evaluation metrics	
AUC	0.9509119
Kappa	0.235036
Sensitivity	0.9556962
Specificity	0.8460194
CCR	0.8492841
HL	1.9422848 ns

Aquila chrysaetos

Pairwise correlations, spatial descriptor and FDR

Human, lithology and land use variables were not correlated, thus all of them were selected in pairwise correlations.

Variables selected			
**Climate**	**Topography**	**Spatial**	**Variables excluded by the FDR**
DP01Win	Alt	Y	WE
DP30Spr	DAlt	X	Gyps
DTn0Sum	SE	XY	U500
DTn20Aut	WE	X^2	MF
TSum	CTI	X^3	SE
TRan		Y^2	
TnWin		Y^3	
AET		X^2Y	
		XY^2	

Final favourability model

Variables	ß	SE	Sig
(Intercept)	5.57E+00	1.72E+00	**
CTI	-5.37E-01	9.21E-02	***
TRan	3.63E-01	7.22E-02	***
Shrub	2.38E+00	2.22E-01	***
Alt	6.41E-04	2.26E-04	**
DAlt	1.55E-03	2.10E-04	***
Spatial	6.38E-01	9.12E-02	***
DP30Spr	-4.33E-01	7.61E-02	***
DHi	6.83E-03	1.74E-03	***
BLF	1.99E+00	3.38E-01	***
Past	-4.39E+00	1.22E+00	***
CF	1.27E+00	3.03E-01	***
Clay	-0.5084866	0.1495641	***
HPd	-0.0011865	0.000636	ns
AET	-0.0009261	0.0003611	*
Grav	-0.4254798	0.2645641	ns
TnWin	0.2504006	0.0678657	***
TSum	-0.1988456	0.0703953	**
DTn20Aut	-0.0401276	0.0191527	*

Evaluation metrics	
AUC	0.8345716
Kappa	0.4719186
Sensitivity	0.7692308
Specificity	0.7506717
CCR	0.7562170
HL	28.4659447 ***

Lagopus mutus

Pairwise correlations, spatial descriptor and FDR

Human, lithology and land use variables were not correlated, thus all of them were selected in pairwise correlations.

Variables selected			
**Climate**	**Topography**	**Spatial**	**Variables excluded by the FDR**
DP10Spr	Alt	Y	Past
DTn0	SE	XY	Calc
DTn0Sum	WE	X^2	WE
DTn20	CTI	X^3	MF
TSum		Y^3	
TRan		XY^2	
AET			

Final favourability model

Variables	ß	SE	Sig
(Intercept)	-1.09E+01	3.23E+00	***
Spatial	4.61E-01	1.81E-01	*
DTn0	5.79E-02	1.53E-02	***
DP10Spr	1.85E-01	1.22E-01	ns
Clay	-1.15E+01	6.38E+00	ns
HPd	-3.77E-01	2.80E-01	ns

Evaluation metrics	
AUC	0.9994035
Kappa	0.7386766
Sensitivity	1
Specificity	0.9918033
CCR	0.9918990
HL	10.9099107 ns

Tetrao urogallus

Pairwise correlations, spatial descriptor and FDR

Human, lithology and land use variables were not correlated, thus all of them were selected in pairwise correlations.

Variables selected			
**Climate**	**Topography**	**Spatial**	**Variables excluded by the FDR**
DP10Spr	Alt	Y	SE
DP10Sum	SE	X	Calc
DTn0Sum	WE	XY	WE
DTn20	CTI	X^2	
Tann		X^3	
TRan		Y^2	
AET		X^2Y	
		XY^2	

Final favourability model

Variables	ß	SE	Sig
(Intercept)	2.57E+01	9.73E+00	**
CTI	-2.21E+00	5.55E-01	***
Spatial	7.09E-01	1.25E-01	***
CF	1.10E+01	1.68E+00	***
U500	2.57E-02	4.15E-03	***
TRan	3.29E-01	1.20E-01	**
AET	-1.29E-02	4.41E-03	**
BLF	8.13E+00	1.45E+00	***
Sil	-2.39E+00	5.62E-01	***
MF	7.24E+00	1.61E+00	***
Shrub	6.44E+00	1.45E+00	***
Alt	4.13E-03	8.59E-04	***
DTn0Sum	-0.9831937	0.3410507	**
Past	6.9874094	3.1664896	*
Grav	-6.5479217	4.8726068	ns

Evaluation metrics	
AUC	0.9952019
Kappa	0.5690619
Sensitivity	0.9793103
Specificity	0.9616502
CCR	0.9621326
HL	8.3280171 ns

Perdix perdix

Pairwise correlations, spatial descriptor and FDR

Human, lithology and land use variables were not correlated, thus all of them were selected in pairwise correlations.

Variables selected			
**Climate**	**Topography**	**Spatial**	**Variables excluded by the FDR**
DP10Spr	Alt	Y	SE
DP30Sum	SE	XY	WE
DTn0Sum	WE	X^2	
DTn20	CTI	X^3	
TSum		Y^2	
TRan		Y^3	
TxWin		X^2Y	
SIDSum		XY^2	
AET			

Final favourability model

Variables	ß	SE	Sig
(Intercept)	2.92E+01	6.72E+00	***
CTI	-2.01E+00	3.17E-01	***
Spatial	4.77E-01	8.68E-02	***
AET	-1.13E-02	2.56E-03	***
DTn20	-1.67E-01	7.06E-02	*
BLF	5.62E+00	9.43E-01	***
Shrub	5.16E+00	9.32E-01	***
Alt	4.36E-03	1.01E-03	***
Past	5.13E+00	1.68E+00	**
MF	3.16E+00	1.26E+00	*
CF	2.68E+00	9.51E-01	**
U100	-1.42E-02	5.70E-03	*
TxWin	0.366997	0.162182	*
DTn0Sum	0.585695	0.257855	*
DHi	0.016267	0.007717	*
DP30Sum	-0.618032	0.255162	*

Evaluation metrics	
AUC	0.9891759
Kappa	0.5682411
Sensitivity	0.9686099
Specificity	0.9431662
CCR	0.9442351
HL	10.7791335 ns

Otis tarda

Pairwise correlations, spatial descriptor and FDR

Human, lithology and land use variables were not correlated, thus all of them were selected in pairwise correlations.

Variables selected			
**Climate**	**Topography**	**Spatial**	**Variables excluded by the FDR**
DP30Spr	Alt	Y	U100
DP30Sum	Slop	X	Calc
DTn0Sum	SE	XY	DHi
DTn20Aut	WE	X^2	
TRan		X^3	
TxSpr		X^2Y	
SIDSum		XY^2	
SISWin			
ROff			
AET			

Final favourability model

Variables	ß	SE	Sig
(Intercept)	-7.15E+00	1.74E+00	***
Slop	-7.61E-01	8.38E-02	***
NIAL	2.84E+00	2.19E-01	***
SIDSum	5.07E-01	3.01E-01	ns
Spatial	4.08E-01	8.14E-02	***
CF	-5.10E+00	1.36E+00	***
TRan	1.97E-01	5.05E-02	***
HPd	-1.80E-03	7.60E-04	*
Gyps	-4.07E+00	2.26E+00	ns
AET	-1.80E-03	5.90E-04	**
DTn20Aut	7.27E-02	2.88E-02	*
DTn0Sum	-2.62E+00	1.27E+00	*
U500	0.0024771	0.0010579	*
WE	0.0079399	0.0054528	ns

Evaluation metrics	
AUC	0.9215608
Kappa	0.3883926
Sensitivity	0.8863636
Specificity	0.7981172
CCR	0.8068953
HL	5.4435670 ns

Picus viridis

Pairwise correlations, spatial descriptor and FDR

Human, lithology and land use variables were not correlated, thus all of them were selected in pairwise correlations.

Variables selected			
**Climate**	**Topography**	**Spatial**	**Variables excluded by the FDR**
DP1Sum	Alt	X	HPd
DP30Sum	DAlt	XY	NIAL
DTn0Sum	SE	X^2	Clay
DTn20	WE	Y^2	
PSpr	CTI	Y^3	
TAut		X^2Y	
TRan		XY^2	
AET			

Final favourability model

Variables	ß	SE	Sig
(Intercept)	-7.51E+00	1.87E+00	***
Spatial	8.82E-01	7.67E-02	***
DAlt	1.87E-03	2.86E-04	***
DTn20	-3.20E-02	4.47E-03	***
DTn0Sum	-1.36E+00	2.08E-01	***
BLF	3.15E+00	5.14E-01	***
PSpr	-2.86E-03	9.88E-04	**
MF	3.67E+00	9.30E-01	***
CF	1.82E+00	4.73E-01	***
DHi	-8.08E-03	2.19E-03	***
Shrub	1.09E+00	2.64E-01	***
Alt	1.76E-03	3.35E-04	***
SE	0.0097427	0.0031684	**
U500	-0.0014572	0.0008354	ns
Gyps	2.0835307	0.7763705	**
TAut	0.1962981	0.0604723	**
U100	-0.0036382	0.0015055	*
CTI	0.1801179	0.0690682	**
Sil	0.4071759	0.1650496	*
Calc	0.3333775	0.1835942	ns

Evaluation metrics	
AUC	0.8369539
Kappa	0.4148709
Sensitivity	0.7716463
Specificity	0.7686796
CCR	0.7711002
HL	24.7656540 **

Pyrrhocorax graculus

Pairwise correlations, spatial descriptor and FDR

Human, lithology and land use variables were not correlated, thus all of them were selected in pairwise correlations.

Variables selected			
**Climate**	**Topography**	**Spatial**	**Variables excluded by the FDR**
DTn0Sum	Alt	Y	U500
DTn20	SE	XY	SE
PSpr	WE	X^2	Shrub
TJul	CTI	Y^2	WE
TRan		Y^3	
TxWin		X^2Y	
AET		XY^2	

Final favourability model

Variables	ß	SE	Sig
(Intercept)	3.38E+01	4.96E+00	***
Spatial	4.63E-01	8.37E-02	***
CTI	-1.88E+00	2.70E-01	***
Calc	3.42E+00	4.01E-01	***
TJul	-6.38E-01	1.16E-01	***
DTn0Sum	9.52E-01	3.32E-01	**
CF	-3.10E+00	8.84E-01	***
TRan	1.77E-01	7.33E-02	*
AET	7.33E-03	3.54E-03	*
PSpr	-3.06E-03	1.79E-03	ns

Evaluation metrics	
AUC	0.9896141
Kappa	0.5892420
Sensitivity	0.9879518
Specificity	0.9399091
CCR	0.9421628
HL	9.9494821 ns

Pyrrhocorax pyrrhocorax

Pairwise correlations, spatial descriptor and FDR

Human, lithology and land use variables were not correlated, thus all of them were selected in pairwise correlations.

Variables selected			
**Climate**	**Topography**	**Spatial**	**Variables excluded by the FDR**
DP01Win	Alt	Y	WE
DP10Sum	DAlt	XY	Past
DTn0Sum	SE	X^2	AET
DTn20Aut	WE	X^3	TRan
PSpr	CTI	Y^2	DP01Win
TAut		X^2Y	Clay
TRan		XY^2	
AET			

Final favourability model

Variables	ß	SE	Sig
(Intercept)	1.35E+01	1.79E+00	***
Spatial	9.11E-01	4.72E-02	***
DAlt	1.61E-03	2.08E-04	***
Shrub	1.34E+00	2.34E-01	***
HPd	-9.40E-04	4.01E-04	*
SE	-1.32E-02	2.91E-03	***
PSpr	-3.90E-03	7.92E-04	***
DTn20Aut	-1.02E-01	2.00E-02	***
CTI	-6.27E-01	9.20E-02	***
CF	-1.57E+00	3.06E-01	***
U100	6.33E-03	1.49E-03	***
TAut	-1.23E-01	2.29E-02	***
DP10Sum	-0.1288606	0.0429071	**
DHi	0.005302	0.0020781	*
U500	-0.0014488	0.0007078	*
Calc	0.4651237	0.1302131	***
Grav	0.6346786	0.2509335	*

Evaluation metrics	
AUC	0.8667009
Kappa	0.5460969
Sensitivity	0.8204986
Specificity	0.7627748
CCR	0.7824039
HL	26.6660089 ***

Galemys pyrenaicus

Pairwise correlations, spatial descriptor and FDR

Human, lithology and land use variables were not correlated, thus all of them were selected in pairwise correlations.

Variables selected			
**Climate**	**Topography**	**Spatial**	**Variables excluded by the FDR**
DP30Sum	Alt	X	SE
DTn0Sum	DAlt	XY	WE
DTn20	SE	X^2	
TRan	WE	X^3	
SIDSpr	CTI	Y^2	
PET		Y^3	
AET		X^2Y	

Final favourability model

Variables	ß	SE	Sig
(Intercept)	2.55E+00	3.27E+00	ns
SIDSpr	-1.18E+00	3.93E-01	**
PET	-1.51E-02	1.97E-03	***
Spatial	4.74E-01	8.02E-02	***
DAlt	1.93E-03	3.01E-04	***
U100	-1.73E-02	2.88E-03	***
Calc	2.47E+00	1.32E+00	ns
DTn20	6.08E-02	1.61E-02	***
NIAL	-1.42E+00	4.84E-01	**
Clay	4.07E+00	1.32E+00	**
DP30Sum	-3.26E-01	1.50E-01	*
TRan	1.07E-01	4.91E-02	*
BLF	1.1594021	0.4496624	**
MF	1.5278942	0.5935913	*
Shrub	0.9421291	0.4291355	*
CTI	0.2898229	0.1644539	ns
DHi	0.0058643	0.0034182	ns
Sil	3.4491757	1.3114712	**
Grav	3.4589674	1.3913402	*

Evaluation metrics	
AUC	0.9451618
Kappa	0.4641254
Sensitivity	0.9259259
Specificity	0.8375391
CCR	0.8460814
HL	8.0409874 ns

Canis lupus

Pairwise correlations, spatial descriptor and FDR

Human, lithology and land use variables were not correlated, thus all of them were selected in pairwise correlations.

Variables selected			
**Climate**	**Topography**	**Spatial**	**Variables excluded by the FDR**
DP30Win	Alt	Y	Shrub
DP30Sum	SE	X	CF
DTn0Sum	WE	XY	DHi
DTn20	CTI	X^2	Clay
TRan		X^3	WE
TnJul		Y^2	
SIDWin		Y^3	
ROff		X^2Y	
AET		XY^2	

Final favourability model

Variables	ß	SE	Sig
(Intercept)	7.81E-01	1.06E+00	ns
Spatial	9.86E-01	5.03E-02	***
TnJul	-4.44E-01	6.54E-02	***
HPd	-3.51E-03	1.06E-03	***
Past	-1.91E+00	7.94E-01	*
AET	1.42E-03	6.67E-04	*
SIDWin	1.52E+00	4.82E-01	**
ROff	5.48E-04	2.49E-04	*
DP30Sum	2.88E-01	1.77E-01	ns
U500	1.87E-03	1.04E-03	ns
TRan	1.37E-01	4.17E-02	**
Alt	-9.77E-04	3.45E-04	**
DTn20	0.029119	0.0096646	**
BLF	0.997867	0.4280201	*
MF	1.1523212	0.5657367	*

Evaluation metrics	
AUC	0.9549853
Kappa	0.7057846
Sensitivity	0.9214418
Specificity	0.8833412
CCR	0.8911078
HL	64.9676153 ***

Lutra lutra

Pairwise correlations, spatial descriptor and FDR

Human, lithology and land use variables were not correlated, thus all of them were selected in pairwise correlations.

Variables selected			
**Climate**	**Topography**	**Spatial**	**Variables excluded by the FDR**
DP1Win	Alt	Y	SE
DP30Spr	DAlt	X	
DP30Sum	SE	XY	
DTn0Sum	WE	X^2	
DTn20	CTI	Y^2	
DTx25Spr		Y^3	
TRan		X^2Y	
TnSum		XY^2	
SIDWin			
AET			

Final favourability model

Variables	ß	SE	Sig
(Intercept)	-4.69E+00	1.50E+00	**
Spatial	9.38E-01	6.13E-02	***
NIAL	-7.91E-01	1.73E-01	***
U500	5.55E-03	7.58E-04	***
AET	3.43E-03	3.94E-04	***
TRan	1.53E-01	2.92E-02	***
CF	1.77E+00	3.24E-01	***
Shrub	1.29E+00	2.45E-01	***
Grav	9.60E-01	2.19E-01	***
DAlt	1.43E-03	2.07E-04	***
DP30Spr	-2.73E-01	8.14E-02	***
Alt	-1.23E-03	2.58E-04	***
SIDWin	-1.1192614	0.243527	***
U100	0.0054707	0.0015113	***
Clay	0.5139878	0.1405211	***
HPd	-0.0003003	0.0001439	*
BLF	1.0727376	0.3473141	**
DP30Sum	-0.3856548	0.1161857	***
DHi	0.005279	0.0019783	**
DP1Win	-0.0161865	0.0106114	ns
CTI	0.1195982	0.0709648	ns
DTn20	0.012441	0.0048466	*
TnSum	-0.0866395	0.0424098	*
Gyps	0.4284977	0.2949751	ns

Evaluation metrics	
AUC	0.8389843
Kappa	0.5040501
Sensitivity	0.7481600
Specificity	0.7654604
CCR	0.7552751
HL	19.0502509 *

Ursus arctos

Pairwise correlations, spatial descriptor and FDR

Human, lithology and land use variables were not correlated, thus all of them were selected in pairwise correlations.

Variables selected			
**Climate**	**Topography**	**Spatial**	**Variables excluded by the FDR**
DP30Spr	Alt	Y	CF
DP30Sum	SE	X	SE
DTn0Spr	WE	XY	WE
DTn0Sum	CTI	X^3	
DTn20		Y^2	
TJul		Y^3	
TRan		X^2Y	
SIDSum		XY^2	
ROff			
AET			

Final favourability model

Variables	ß	SE	Sig
(Intercept)	6.66E+00	5.68E+00	ns
Spatial	7.20E-01	8.85E-02	***
HPd	-5.78E-02	3.07E-02	ns
U500	1.41E-02	2.29E-03	***
DHi	3.12E-02	6.82E-03	***
Past	6.75E+00	1.58E+00	***
Clay	1.45E+00	6.28E-01	*
ROff	1.54E-03	4.67E-04	***
DTn0Sum	-9.84E-01	3.25E-01	**
Grav	3.26E+00	1.29E+00	*
Shrub	3.54E+00	9.30E-01	***
DTn0Spr	8.02E-02	1.60E-02	***
BLF	2.2724366	0.9538473	*
MF	3.6255715	1.2373222	**
NIAL	3.6905897	1.2625289	**
CTI	-0.9455483	0.3461675	**

Evaluation metrics	
AUC	0.9909335
Kappa	0.5785035
Sensitivity	0.9805825
Specificity	0.9482556
CCR	0.9495102
HL	10.6984415 ns

Felis silvestris

Pairwise correlations, spatial descriptor and FDR

Human, lithology and land use variables were not correlated, thus all of them were selected in pairwise correlations.

Variables selected			
**Climate**	**Topography**	**Spatial**	**Variables excluded by the FDR**
DP10Sum	Alt	Y	Sil
DTn0Sum	DAlt	X	U100
DTn20	SE	XY	MF
PSpr	WE	X^2	TRan
TSpr	CTI	X^3	Gyps
TRan		Y^2	U500
AET		Y^3	
		X^2Y	
		XY^2	

Final favourability model

Variables	ß	SE	Sig
(Intercept)	-1.19E+00	3.54E-01	***
DAlt	1.25E-03	1.46E-04	***
Spatial	1.04E+00	7.87E-02	***
Shrub	1.42E+00	2.04E-01	***
Alt	6.03E-04	1.24E-04	***
BLF	2.42E+00	3.19E-01	***
PSpr	-3.83E-03	8.33E-04	***
AET	2.07E-03	3.25E-04	***
CF	7.09E-01	2.80E-01	*
Past	-1.84E+00	7.17E-01	*
NIAL	-4.52E-01	1.88E-01	*
DTn20	-7.30E-03	3.31E-03	*
DP10Sum	-0.0773781	0.0327702	*
Calc	0.2352353	0.1088029	*
Grav	0.3993633	0.1893693	*
HPd	-0.0002846	0.0001843	ns
WE	-0.0055291	0.0030949	ns

Evaluation metrics	
AUC	0.7683479
Kappa	0.3701999
Sensitivity	0.6823266
Specificity	0.7107955
CCR	0.7012057
HL	8.8091032 ns

Lynx pardinus

Pairwise correlations, spatial descriptor and FDR

Human, lithology and land use variables were not correlated, thus all of them were selected in pairwise correlations.

Variables selected			
**Climate**	**Topography**	**Spatial**	**Variables excluded by the FDR**
DP30Spr	Alt	Y	Gyps
DP30Sum	DAlt	X	Shrub
DTn0Spr	SE	XY	Sil
DTn0Sum	WE	X^2	TRan
DTn20	CTI	X^3	Clay
DTx25Aut		Y^2	WE
TRan		Y^3	MF
SISSum		X^2Y	BLF
ROff		XY^2	AET
AET			HPd

Final favourability model

Variables	ß	SE	Sig
(Intercept)	-2.55E+02	7.29E+01	***
Spatial	3.50E-01	2.03E-01	ns
DTx25Aut	2.74E-01	7.83E-02	***
CF	6.14E+00	1.89E+00	**
NIAL	-5.51E+00	2.21E+00	*
SISSum	3.12E+01	9.45E+00	***
DP30Sum	1.44E+01	3.70E+00	***
DP30Spr	-2.28E+00	9.13E-01	*
ROff	1.02E-02	4.43E-03	*
Grav	-1.84E+00	9.69E-01	ns

Evaluation metrics	
AUC	0.9886930
Kappa	0.1567660
Sensitivity	0.9655172
Specificity	0.9465808
CCR	0.9466843
HL	9.6965038 ns

Cervus elaphus

Pairwise correlations, spatial descriptor and FDR

Human, lithology and land use variables were not correlated, thus all of them were selected in pairwise correlations.

Variables selected			
**Climate**	**Topography**	**Spatial**	**Variables excluded by the FDR**
DP10Spr	Alt	Y	Gyps
DP30Sum	DAlt	X	Calc
DTn0Sum	SE	XY	WE
DTn20Aut	WE	X^2	DP30Sum
TRan	CTI	X^3	MF
TnJan		Y^2	
TxJul		Y^3	
AET		X^2Y	

Final favourability model

Variables	ß	SE	Sig
(Intercept)	-9.88E+00	7.68E-01	***
U100	1.17E-02	1.43E-03	***
Spatial	7.20E-01	7.44E-02	***
BLF	3.40E+00	3.14E-01	***
CF	2.87E+00	2.93E-01	***
Shrub	1.83E+00	2.09E-01	***
TxJul	1.38E-01	2.25E-02	***
DTn0Sum	8.46E-01	1.56E-01	***
DP10Spr	9.47E-02	2.23E-02	***
U500	3.92E-03	7.44E-04	***
SE	8.49E-03	2.85E-03	**
DAlt	3.00E-04	1.41E-04	*
Sil	-0.2496909	0.1045836	*
AET	0.0008928	0.0003652	*
HPd	0.0001989	0.0001129	ns
TRan	0.0692817	0.0337668	*
DTn20Aut	0.0304919	0.0182856	ns

Evaluation metrics	
AUC	0.8060400
Kappa	0.4235189
Sensitivity	0.7223899
Specificity	0.7362366
CCR	0.7319141
HL	10.2530292 ns

Rupicapra pyrenaica

Pairwise correlations, spatial descriptor and FDR

Human, lithology and land use variables were not correlated, thus all of them were selected in pairwise correlations.

Variables selected

Climate	Topography	Spatial	Variables excluded by the FDR
DP10Spr	Alt	X	Shrub
DP10Sum	SE	XY	Gyps
DTn0Sum	WE	X^2	Calc
DTn20	CTI	X^3	WE
Tann		Y^2	
TRan		Y^3	
AET		X^2Y	
		XY^2	

Final favourability model

Variables	ß	SE	Sig
(Intercept)	2.86E+01	6.22E+00	***
Spatial	4.15E-01	8.37E-02	***
Alt	3.05E-03	6.14E-04	***
CTI	-2.19E+00	3.67E-01	***
U100	2.08E-02	7.21E-03	**
DHi	1.84E-02	9.35E-03	*
DP10Sum	2.03E-01	1.10E-01	ns
DTn0Sum	7.48E-01	4.18E-01	ns

Evaluation metrics	
AUC	0.9952173
Kappa	0.6543732
Sensitivity	0.9770642
Specificity	0.9603143
CCR	0.9610023
HL	5.3400624 ns

Capra pyrenaica

Pairwise correlations, spatial descriptor and FDR

Human, lithology and land use variables were not correlated, thus all of them were selected in pairwise correlations.

Variables selected			
**Climate**	**Topography**	**Spatial**	**Variables excluded by the FDR**
DP01Win	Alt	Y	DTx25Spr
DP30Spr	DAlt	X	MF
DP30Sum	SE	XY	SE
DTn0Sum	WE	X^2	Clay
DTn20	CTI	X^3	AET
DTx25Spr		Y^2	DP30Sum
TRan		Y^3	
TnWin		X^2Y	
SISWin		XY^2	
AET			

Final favourability model

Variables	ß	SE	Sig
(Intercept)	2.07E+00	2.72E+00	ns
Spatial	1.22E+00	5.74E-02	***
CTI	-4.28E-01	1.48E-01	**
DP30Spr	9.93E-01	1.17E-01	***
DAlt	1.87E-03	2.91E-04	***
Shrub	1.86E+00	3.35E-01	***
TRan	2.57E-01	4.02E-02	***
DHi	1.68E-02	3.29E-03	***
CF	1.10E+00	4.36E-01	*
WE	-1.24E-02	5.09E-03	*
Grav	8.07E-01	3.73E-01	*
NIAL	-9.52E-01	4.06E-01	*
DTn20	-0.0086115	0.004401	ns
Gyps	-2.2376372	1.5361969	ns
U100	-0.0037727	0.0025533	ns

Evaluation metrics	
AUC	0.9376692
Kappa	0.5036426
Sensitivity	0.9088099
Specificity	0.8341558
CCR	0.843255
HL	7.4210190 ns
